# Bis‐Alkoxide Dysprosium(III) Crown Ether Complexes Exhibit Tunable Air Stability and Record Energy Barrier

**DOI:** 10.1002/advs.202308548

**Published:** 2024-02-23

**Authors:** Wen‐Jie Xu, Qian‐Cheng Luo, Zi‐Han Li, Yuan‐Qi Zhai, Yan‐Zhen Zheng

**Affiliations:** ^1^ Department of Hepatobiliary Surgery and Institute of Advanced Surgical Technology and Engineering The First Affiliated Hospital of Xi'an Jiaotong University Xi'an Shaanxi 710061 P. R. China; ^2^ Frontier Institute of Science and Technology (FIST) State Key Laboratory of Electrical Insulation and Power Equipment MOE Key Laboratory for Nonequilibrium Synthesis and Modulation of Condensed Matter Xi'an Key Laboratory of Electronic Devices and Material Chemistry, and School of Chemistry Xi'an Jiaotong University Xi'an Shaanxi 710054 P. R. China

**Keywords:** air‐stability, dysprosium, energy barrier, hexagonal‐bipyramidal, single‐molecule magnets

## Abstract

High‐performance and air‐stable single‐molecule magnets (SMMs) can offer great convenience for the fabrication of information storage devices. However, the controversial requisition of high stability and magnetic axiality is hard to balance for lanthanide‐based SMMs. Here, a family of dysprosium(III) crown ether complexes possessing hexagonal‐bipyramidal (pseudo‐D_6h_ symmetry) local coordination geometry with tunable air stability and effective energy barrier for magnetization reversal (*U*_eff_) are shown. The three complexes share the common formula of [Dy(18‐C‐6)L_2_][I_3_] (18‐C‐6 = 1,4,7,10,13,16‐hexaoxacyclooctadecane; L = I, **1**; L = O^t^Bu **2** and L = 1‐AdO **3**). **1** is highly unstable in the air. **2** can survive in the air for a few minutes, while **3** remains unchanged in the air for more than 1 week. This is roughly in accordance with the percentage of buried volumes of the axial ligands. More strikingly, **2** and **3** show progressive enhancement of *U*_eff_ and **3** exhibits a record high *U*_eff_ of 2427(19) K, which significantly contributes to the 100 s blocking temperature up to 11 K for Yttrium‐diluted sample, setting a new benchmark for solid‐state air‐stable SMMs.

## Graphical abstract

Two bis‐alkoxide Dy(III)‐18‐Crown‐6 single‐molecule magnets with local hexagonal‐bipyramidal coordination geometry have been isolated by using the triiodide precursor and show progressive enhancement of magnetic axiality and air stability. The adamantanol analog exhibits a record *U*_eff_ of 2427(19) K and the *T*_B_^100s^ of 11 K, setting a new benchmark for solid‐state air‐stable SMMs.

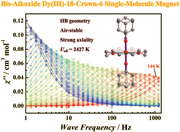

## 1 Introduction

Molecular systems possessing magnetic bistability and discontinuous energy levels are termed single‐molecule magnets (SMMs),^[^ ^1^ ^]^ which, in addition to exhibiting fundamental potential for ultra‐high density data storage,^[^ ^2^ ^]^ have inspired proposals for applications in molecular spintronics^[^ ^3^ ^]^ and quantum information processing.^[^ ^4^ ^]^ The characteristics of high‐performance SMMs are the high effective energy barrier (*U*_eff_) to magnetization reversal, high blocking temperature (*T*_B_), and large coercive field. Indeed, *U*_eff_ is derived from the high‐temperature Orbach process and can be determined experimentally;^[^ ^5^ ^]^ *T*_B_ has been traditionally defined as the temperature where the relaxation time reaches 100 seconds (*T*_B_^100s^) for superparamagnetic materials.^[^ ^6^ ^]^ There are other definitions of *T*_B_, such as the peak temperature of the zero‐field cooled magnetization (*T*_B_^ZFC^) and the observable temperature of magnetic hysteresis loops (*T*_B_^hys^).^[^ ^6^, ^7^ ^]^

Dysprosium(III) is the most investigated ion for high‐performance SMMs due to its ^6^H_15/2_ ground‐state term with large orbital momentum.^[^ ^8^ ^]^ In addition, the magnetic anisotropy for Dy^3+^ is more controllable due to the oblate shape of 4*f* electron densities for *M*_J_ = ±15/2, which requires strong axial a ligand field to create magnetic axiality.^[^ ^9^ ^]^ Thus, single‐point coordinate SMMs with pentagonal‐bipyramidal (PB) geometry^[^ ^10^, ^11^, ^12^, ^13^ ^]^ or sandwiched SMMs with η*^5^*‐bound cyclopentadienyl (Cp) ligands^[^ ^14^ ^−^ ^21^ ^]^ are excelled. More recently, Gould et al. successfully isolated a mixed‐valent dinuclear complex (Cp*^iPr5^*)_2_Dy_2_I_3,_ which exhibits a *U*_eff_ of 2347 K and *T*_B_^100s^ up to 72 K, setting the highest figure‐of‐merit for SMMs.^[^ ^19^ ^]^ However, both PB and Cp‐based dysprosium(III) SMMs are air‐sensitive, especially for the latter family, which belongs to the organometallic compounds.

Usually, air‐stable Dy(III) complexes require a least coordination number (CN) of 7 due to the large radius of the Dy(III) ion.^[^ ^22^ ^]^ Hence, some PB Dy(III)‐SMMs could be air‐stable, but their *U*_eff_ is usually smaller than 1200 K (**Table** **1**).^[^ ^23^, ^24^, ^25^, ^26^ ^]^ Other higher‐performing PB Dy(III)‐SMMs are highly air‐sensitive, presumably due to the straighter axial X─Dy─X coordination angles.^[^ ^10^, ^11^, ^12^, ^13^ ^]^ If CN is raised to eight, the stability of the Dy(III) complexes is much enhanced.^[^ ^22^ ^]^ However, to retain the magnetic axiality, only the hexagonal‐bipyramidal (HB) coordination geometry is preferred for CN = 8.^[^ ^9^, ^27^, ^28^, ^29^, ^30^, ^31^ ^]^ So far, this practice has been represented by the complex *RRRR*‐Dy‐*D*_6h_F_12_, which displays a *U*_eff_ of 1833 K and *T*_B_^100s^ of 5 K.^[^ ^31^ ^]^ This complex takes advantage of the electron‐withdrawing effect on the equatorial coordination plane of the HB geometry, which enlightens us to further weaken the basicity of the equatorial ligand for higher performance of Dy(III)‐SMMs with pseudo‐*D*_6h_ local symmetry.

**Table 1:** Selected high‐performance air‐stable Dy^3+^ SMMs.

Compound	Geometry	*U_eff_* [K]	*T*_B_^100s^ [K]	Ref.
[Dy(Cy_3_PO)_2_(H_2_O)_5_]Br_3_	PB	543	–	[23]
[Dy(*^t^* BuPO(NH*^i^* Pr)_2_)_2_(H_2_O)_5_]I_3_	PB	735	2.4^a)^	[24]
[Dy(bbpen)Br]	PB	1025	3.3^a)^	[25]
[Dy^III^(L^N5^)(Ph_3_SiO)_2_](BPh_4_)	PB	1108	–	[26]
[Dy^III^(L^N6)^(Ph_3_SiO)_2_](Bph_4_)	HB	1124	–	[27]
[Dy^III^(L^E^)(4‐MeO‐PhO)_2_] (BPh_4_)	HB	1338	–	[28]
[Dy^III^L^N6^_R_(L_2_)_2_](BPh_4_)	HB	1453	–	[29]
[Dy(L_1_^N6^)(Ph_3_SiO)_2_][ClO_4_]	HB	1732	–	[30]
*RRRR*‐Dy‐*D*_6h_F_12_	HB	1833	5^b)^	[31]
**3**	HB	2427	10.3^a)^ 11^b)^	This work

^a)^ Extrapolation;

^b)^ Diluted sample.

— Not available above 2 K.

Our previous work showed that the six‐coordinate 1,4,7,10,13,16‐hexaoxacyclooctadecane (18‐C‐6) ligand is an even weaker equatorial ligand compared to the macrocycle schiff bases,^[^ ^32^, ^33^, ^34^ ^]^ especially for the complex [Dy(O^t^Bu)Cl(18‐C‐6)][BPh_4_] with a *U*_eff_ of ca. 1000 K and *T*_B_^hys^ of 4 K, which shows a very flexible structure with temperature‐dependent O─Dy─Cl bonding angles.^[^ ^34^ ^]^ To enhance the structural rigidity, we reason that it is critical to enhance the steric hindrance for the axial ligands so as to fix the axis of the HB geometry. The simplest way is to substitute the chloride with another bulky alkoxide ligand. However, the chemistry is far more complex than we expected. The targeted compound [Dy(18‐C‐6)(O^t^Bu)_2_]^+^ cannot be obtained no matter how we vary the stoichiometry of reagents until we isolated a new triiodide‐based complex [Dy(18‐C‐6)I_2_][I_3_] **1** as the precursor. Two subsequently targeted complexes, namely [Dy(18‐C‐6)(O^t^Bu)_2_][I_3_] **2** and [Dy(18‐C‐6)(1‐AdO)_2_][I_3_] **3**, were then successfully isolated. Moreover, we also found that the stability of the complex follows the steric hindrance of the axial ligands. For **1**, the solid is highly unstable in air; for **2** the crystallinity is quickly lost in a few minutes. Further increasing the steric hindrance by using 1‐adamantanol **3** finally becomes air‐stable. More strikingly, **3** shows the record *U*_eff_ = 2427(19) K, *T*_B_^100s^ = 11 K, and observable open magnetic hysteresis up to 30 K for the yttrium‐diluted sample (vide infra).

## 2 Results and Discussion

### 2.1 Synthesis and Structures

**Scheme 1:**
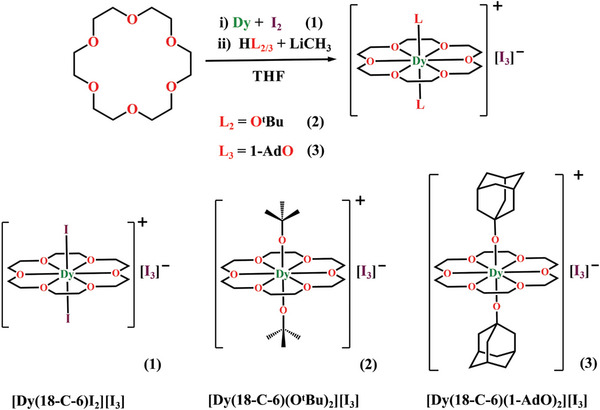
Synthetic routes for **1**–**3**.

The synthesis of compounds **1**–**3** is shown in **Scheme** **1**. It is worth mentioning that without the triiodide precursor, we can only obtain the mono‐alkoxide Dy‐18‐C‐6 complex (i.e., [Dy(O^t^Bu)Cl(18‐C‐6)][BPh_4_]). To the best of our knowledge, this is the first time triiodide has been used as the balanced anion for preparing cationic Dy(III)‐SMMs. Here, the strong peak ≈110 cm^−1^ in the Raman spectra (Figures S1–S3, Supporting Information) demonstrated the presence of I_3_^−^ in **1**–**3**.^[^ ^35^ ^]^ Moreover, using lithium alkoxide rather than sodium or potassium alkoxide to substitute the axial iodide is also crucial, which can avoid the specific binding of 18‐C‐6 to Na^+^ or K^+^ ions. The yttrium‐diluted samples **2@Y** (8.15% Dy based on ICP analysis) and **3@Y** (7.68% Dy based on ICP analysis) were also isolated similarly. Indeed, the isolation of **1** makes the axial ligands on the two sides of the 18‐C‐6 ligand substitutable and fully controllable for this family of HB Dy(III) complexes.

**Figure 1:**
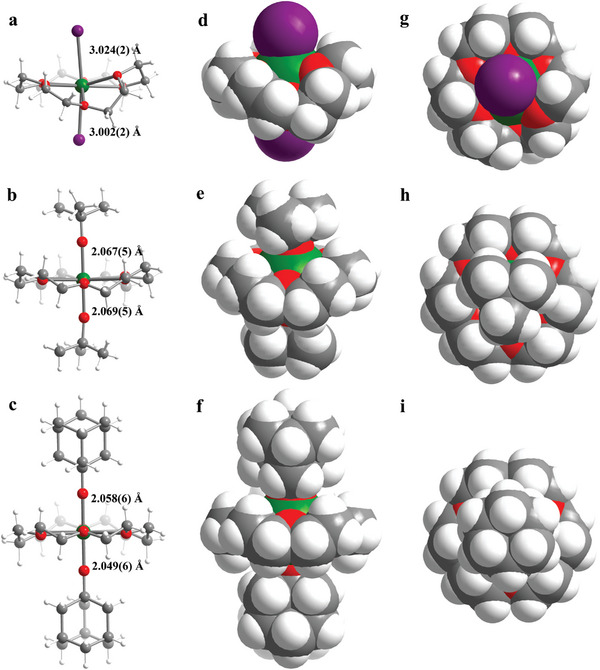
Crystal structures for the [Dy(18‐C‐6)L2]+ cations in **1**–**3** (a–c). Space‐filling models viewed from the side (d–f) and the top (g–i) of **1**–**3**. Color codes: Dy, green; O, red; C, gray; I, purple; H, white.

Complexes **1**–**3** have similar structures (**Figure** **1**). The Dy(III) ion is sitting in the macrocycle and coordinated by six O atoms of the 18‐C‐6 ligand in the equatorial plane. For **1**, the six O atoms of the 18‐C‐6 ligand are more waving than **2** and **3**. This is clearly the effect of steric hindrance of the alkoxide ligands. Also, the two axial I^−^ anions form a more bending I─Dy─I angle of 165.84(3)°, compared to the axial O─Dy─O angle of 178.0(2)° for **2** and 178.0(3)° for **3** (Tables S3, S4, and S7, Supporting Information). Moreover, the average axial Dy─I bond length of 3.013(2) Å for **1** is obviously much longer compared to the average axial Dy─O bond lengths of 2.068(5) Å for **2** and 2.054(6) Å for **3**. Meanwhile, the average equatorial Dy─O bond length of 2.452(9) Å for **1** is shorter than those (2.613(5) Å and 2.636(5) Å) of **2** and **3**. To compare the HB coordination geometry more specifically, the continuous shape measure (CShM) analyses were applied, giving the values of 4.087, 1.019, and 1.077 for **1**, **2**, and **3**, respectively. Compared to the larger values of other geometry, CShM clearly reveals that the Dy^3+^ cation of [Dy(18‐C‐6)L_2_]^+^ is in the HB geometry (Table S10, Supporting Information). It is important to note that the axial Dy─O bond length of **3** is the shortest for Dy(III) compounds with HB geometry up‐to‐date.^[^ ^28^ ^]^ Moreover, the shortest intermolecular Dy∙∙∙Dy distances also remain similar upon cooling, namely 9.31 Å for **2** and 10.14 Å for **3** (Figures S6 and S7, Supporting Information).

The temperature‐dependent single‐crystal structures of **2** and **3** were further characterized to probe the structural rigidity. Upon cooling to 150 K and 100 K, compared to the decreased axial Cl─Dy─O angles of 5° and 10° for A component of [Dy(O^t^Bu)Cl(18‐C‐6)]^+^,^[^ ^34^ ^]^ the axial O─Dy─O angles of **2** and **3** vary only slightly, namely 177.5(1)° and 177.5(2)° for **2** and 177.8(2)° and 177.5(2)° Å for **3** (Tables S5–S6 and S8–S9, Supporting Information). In addition, the CShM values of **2** and **3** at 150 K and 100 K are also very close to those at 298 K (Table S10, Supporting Information), which indicates the enhancement of the rigidity of both molecular structures.

### 2.2 Thermal and Air Stability

**Figure 2:**
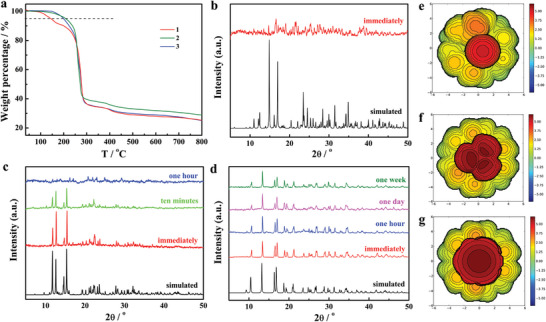
TG analysis of **1**–**3** a), where the 95%‐weight line is shown to determine Td. PXRD spectra of **1**–**3** b–d) in air atmosphere, and the black line is simulated from single crystal data. Topographic steric maps of **1**–**3** e–g), and red and blue indicate the more‐ and less‐hindered zones, respectively.

Thermal properties of **1**–**3** were studied by thermogravimetric analysis (TGA). The decomposition temperatures (*T*_d_, corresponding to a 5% mass loss^[^ ^36^, ^37^ ^]^) of **1**–**3** are 132, 205, and 197 °C, respectively (**Figure** **2**a). Obviously, the structurally rigid **2** and **3** possess good thermal stability. Powder X‐ray diffraction (PXRD) spectra in air atmosphere were obtained to investigate the air stability of **1**–**3** (Figure 2b–d). The immediately measured spectrum after being taken out of the glove box shows no strong diffraction peaks of **1**, indicating it quickly loses crystallinity. In contrast, **2** can last a few minutes before losing its diffraction, while for **3**, the peak position did not change, and no new peak was observed for up to one week, demonstrating its best crystalline stability in air. In addition, the changed infrared spectra, darkened single crystal morphology, and vanishing single‐molecule magnetism of **2** exposed to air for one day indicated its changed molecular structure (Figures S4, S8, S19, and S25, Supporting Information). In contrast, for **3** the time‐dependent infrared spectra, single crystal morphology, and magnetism over time remained consistent, largely indicative of no amorphous decomposition products. (Figures S5, S8, S20, and S26, Supporting Information).

We reason that the differences in air stability directly correlate with the volume of the axial ligands, as indicated by the space‐filling models of the structures (Figure 1d–i). The exposed area of the central Dy^3+^ ions decreases from **1** to **3**. To quantitatively measure the protection extent of the ligands to the central Dy^3+^ ions in **1**–**3**, the percentage of buried volume (%VBur) was used, which can quantify the fraction of the coordination sphere around a metal center occupied by organic ligands.^[^ ^36^, ^37^, ^38^ ^]^ In fact, the %VBur values for **1**–**3** are 43.5%, 53.7%, and 55.7%, respectively, and the increasing feature is clearly shown in the topographic steric maps (Figure 2e–g). Such a steric effect clearly shows that the central Dy^3+^ ion is well protected by the 1‐AdO^−^ ligands of **3**. Hence, the whole molecule of **3** also shows much weaker thermal vibration (Tables S11 and S12, Supporting Information). However, we found that **3** is not stable in polar solution while insoluble in non‐polar solvents, which raises further challenges for making solution stable and neutral SMMs.

### 2.3 Magnetic Properties

Magnetic measurement of **1** shows almost no AC signals under zero DC field due to the weak axial ligand field of the iodide (Figure S12, Supporting Information). Thus, below, we mainly focus on the magnetic properties of **2** and **3**. At 300 K, the *χ*_m_ *T* products (Figures S10a and S11a, Supporting Information) were measured to be 14.09 and 14.10 cm^3^ mol^−1^ K for **2** and **3**, respectively, in agreement with the theoretical value for a free Dy(III) ion (14.17 cm^3^ mol^−1^ K). Upon cooling, the *χ*_m_ *T* values for both complexes slightly decrease before a sharp drop at ca. 14.0 K, suggesting the presence of magnetic blocking. Field‐dependent magnetization curves (*M* vs *H*) at 2 K show the maximum *M* values of 4.95 and 5.10 µ_B_ at 5 T for **2** and **3**, respectively (Figures S10b and S11b, Supporting Information).

**Figure 3:**
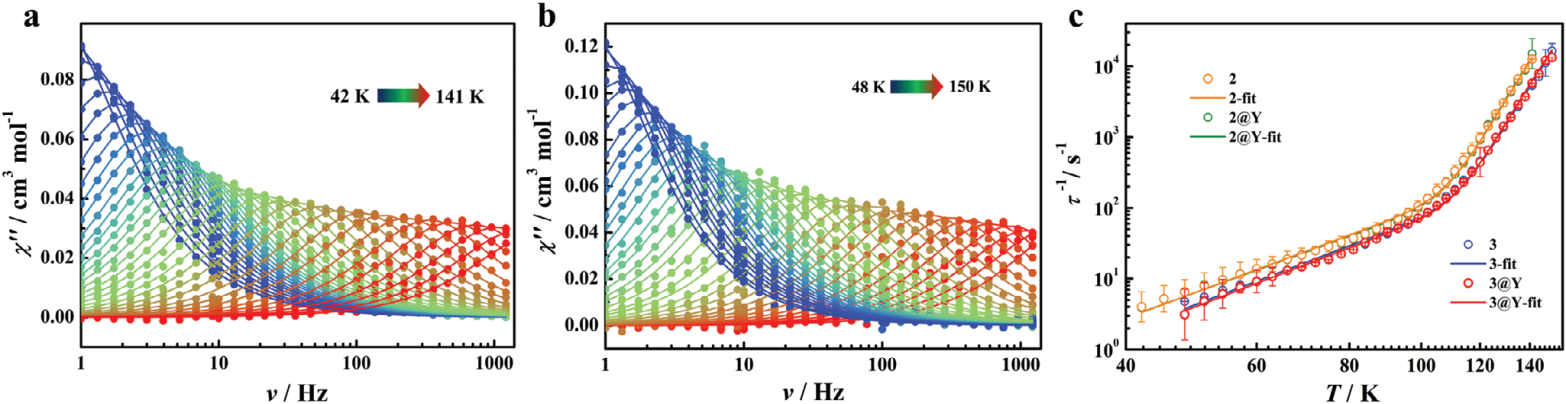
Plots of χ″ vs. v for **2** and **3** a,b) under zero DC field with AC frequencies of 1–1218 Hz. The solid lines best fit with the Debye model. c) Plots of natural log of τ‐1 vs. T for **2**, **2@Y**, **3**, and **3@Y**. The solid line is the best fit with Equation (1).

Temperature‐ and frequency‐dependent AC magnetic susceptibility show slow relaxation of magnetization at zero DC field. The out‐of‐phase component of the AC susceptibility (*χ''*) at the frequency of 1218 Hz shows well‐defined maxima at temperatures up to 138 K for **2** and 144 K for **3**, respectively, indicating high *U*_eff_ (**Figure** **3**a,b; Figures S13–S16, Supporting Information). Temperature‐dependent relaxation times (*τ*) were extracted by fitting the Cole−Cole plots of *χ''* vs. *χ'* using the generalized Debye model (Figures S17 and S18, Supporting Information). The log–log plots of *τ* ^−1^ versus *T* for **2**, **2@Y**, **3**, and **3@Y** all show consistent correlations (Figure 3c). In this temperature regime, the data were fitted by the following equation, considering only the Orbach and Raman processes (Tables S13–S16, Supporting Information).

(1) $$\tau^{-1}={\tau_{0}}^{-1}\;e^{-U_{\mathrm{eff}}/T}+CT^{n}$$

The best‐fitting parameters are given in **Table** **2**. Compound **2**, **2@Y**, **3**, and **3@Y** display *U*_eff_ of 2352(22) K, 2357(26) K, 2427(19) K, and 2429(24) K, respectively. Note that the *U*_eff_ value of **3** is the highest for all known SMMs (Table S17, Supporting Information).

**Table 2:** Fitting parameters for **2**, **2@Y**, **3**, and **3@Y**.

Complex	*U*_eff_ / K	*τ*_0_ / s	*C /* s^−1^ K^−^ *^n^*	*n*
**2**	2352(22)	4.51(2) × 10^−12^	1.56(3) × 10^−6^	3.9(2)
**2@Y**	2357(26)	4.16(3) × 10^−12^	1.99(5) × 10^−6^	3.8(2)
**3**	2427(19)	6.05(2) × 10^−12^	7.10(3) × 10^−7^	4.1(1)
**3@Y**	2429(24)	5.63(3) × 10^−12^	6.95(4) × 10^−7^	4.0(2)

**Figure 4:**
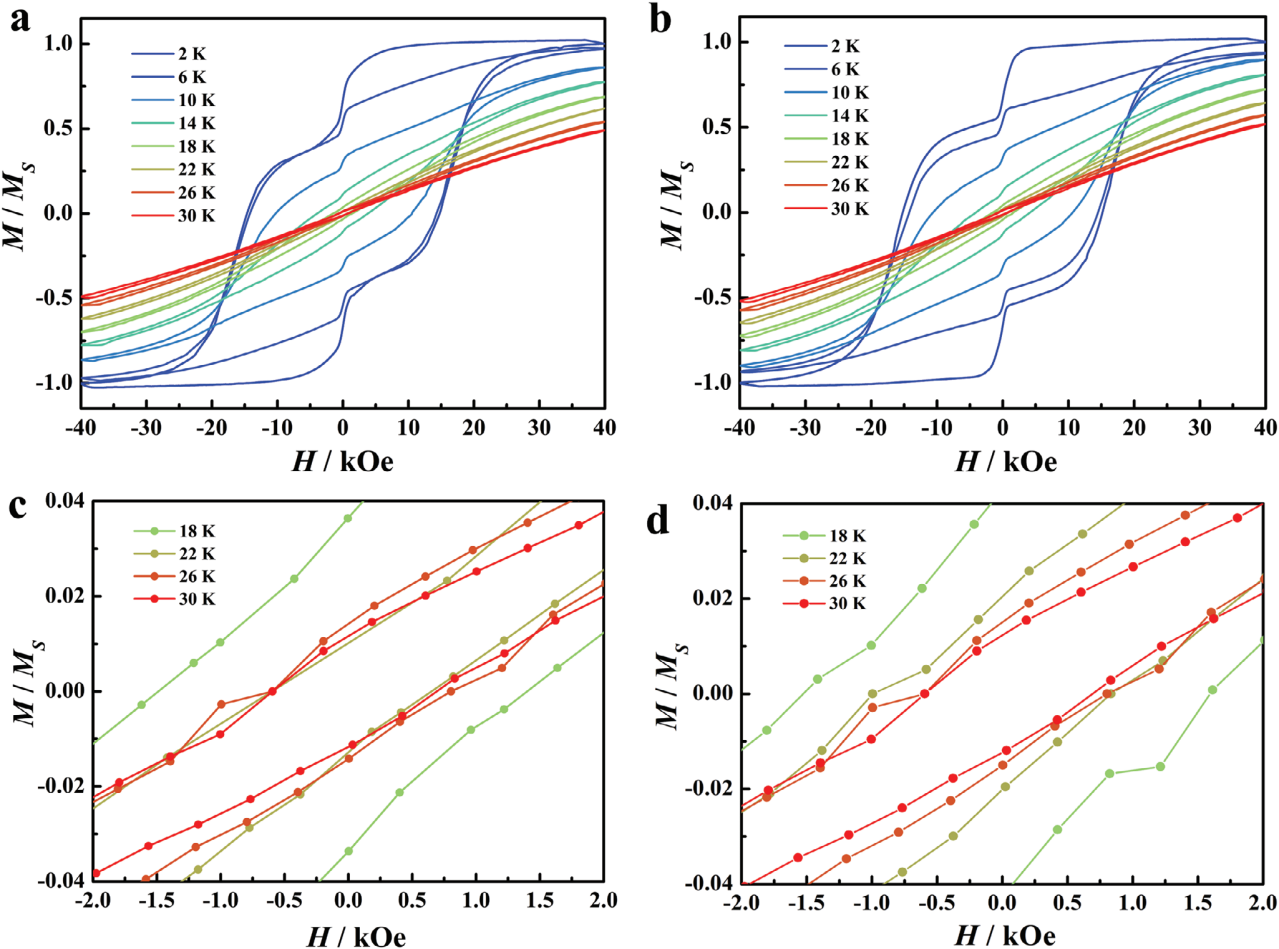
a,b) Magnetic hysteresis loops for **2@Y** and **3@Y** applying an average sweep rate of 200 Oe s‐1. c,d) Magnification of hysteresis loops.

The ZFC curves show the clear peaks at 10(11) K for **2** (**2@Y**), 11 K for **3** (**3@Y**) (Figures S21 and S22, Supporting Information). Applying a sweep rate of 15 Oe s^−1^, the butterfly‐shape hysteresis loops of **2@Y** and **3@Y** show larger coercive fields and remanent moments compared to **2** and **3** (Figures S23 and S24, Supporting Information). Applying a sweep rate of 200 Oe s^−1^, the magnetic hysteresis loops of **2@Y** and **3@Y** both remain open up to 30 K, with coercive fields of ca. 0.6 kOe. (**Figure** **4**). The relaxation times extracted from DC magnetization decay measurements show *T*_B_^100s^ of **2@Y** and **3@Y** are 10 K and 11 K (Figures S27–S28 and Tables S18–S19, Supporting Information).

The slight differences in slow magnetization relaxation of **2** and **3** can be attributed to the similarity in ligand field around the primary coordination sphere of the Dy(III) ions. A bit shorter axial Dy─O bond length and longer equatorial Dy─O bond length of **3** leads to a slightly higher *U*_eff_ value and *T*_B_ than those for **2**.

### 2.4 Ab Initio Calculations

**Figure 5:**
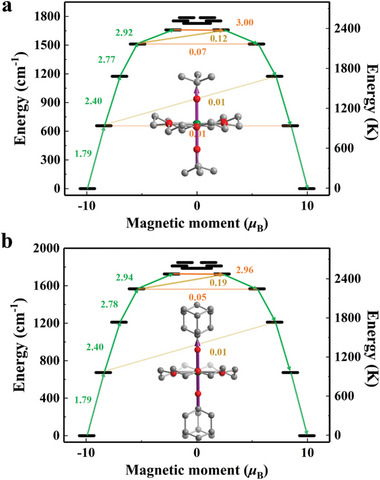
The calculated magnetic relaxation path diagram for **2** and **3** (a,b). The horizontal orange arrows show the QTM/TA‐QTM (thermally‐assisted QTM) processes, while the non‐horizontal green and brown ones represent the spin‐phonon transition paths. The principle magnetic axis of the ground KD of **2** and **3** is highlighted in the purple arrow (insert). All hydrogen atoms are omitted for clarity.

Ab initio calculations at SA‐CASSCF/RASSI level were carried out using OpenMolcas^[^ ^39^ ^]^ to investigate the magnetic relaxation mechanisms of complexes **2** and **3**. Their cationic motifs are directly extracted from their single crystal structures without optimization. As expected, eight Kramers’ doublets (KDs) produced by ^6^H_15/2_ term of Dy(III) ions exhibit very large energy splittings of 2569 K for **2** and 2657 K for **3** (Tables S20 and S21, Supporting Information). The ground KDs in both compounds possess strong axial magnetic anisotropy, which can be reflected in the wavefunction purity of 100 % for *m*_J_ = ±15/2 and their *g*‐tensors approaching Ising limit state with *g*_x_ = *g*_y_ = 0.00, *g*_z_ = 19.89 for **2**, and *g*_x_ = *g*_y_ = 0.00, *g*_z_ = 19.90 for **3**. Moreover, the calculated principle magnetic axes of the ground KD are both along the axial O─Dy─O bonds, which corresponds to the shorter axial Dy─O bonds than equatorial ones. The high axiality can also be observed in the first and second excited KDs with relatively pure |±13/2> and |±11/2> states, which are located at 945 K and 1688 K for **2** and 968 K and 1741 K for **3**, respectively. **Figure** **5** depicts the possible magnetic relaxation pathways, and significant transition moments of 3.00 and 2.96 µ_B_ within the fourth excited KD, for **2** and **3** indicate that strong QTM occurs at this KD, and the theoretical *U*_eff_ values can be evacuated as 2385 K and 2481 K, which are very close to the experimental ones (Tables S23 and S25, Supporting Information). Normally, the crystal field parameters of diagonal $B_{2}^{0}$ and off‐diagonal $B_{2}^{\pm 2}$ terms make the most contribution ondetermining the molecular axiality. Here we utilize the ratio of $|B_{2}^{0}|/\left({|B_{2}^{2}|}^{2}+{|B_{2}^{-2}|}^{2}\right)$ to measure the axiality quantitatively for **2** and **3**,^[^ ^40^ ^]^ the ratios are 698.15 and 3317.46, and higher axiality in the latter complex is correlated with shorter axial Dy─O bonds (Tables S22 and S24, Supporting Information). For comparison, such ratios of the reported complexes *RRRR*‐Dy‐*D*_6h_F_12_ and *SSSS*‐Dy‐*D*_6h_F_12_ were also calculated, and they are merely 43.11 and 84.34, respectively, indicating the combination of axial alkoxide and equatorial 18‐C‐6 ligands can further enhance the uniaxial magnetic anisotropy of Dy(III)‐SMMs.

## 3 Conclusion

In summary, by using the triiodide‐based complex **1** as a precursor we successfully isolated two bis‐alkoxide Dy(III)‐18‐C‐6 complexes **2** and **3** with local hexagonal‐bipyramidal coordination geometries. The sterically more bulky adamantyl group makes the solids of **3** more stable in the air. Strikingly, **3** possesses a record *U*_eff_ = 2427(19) K for reported SMMs up to date, while *T*_B_^100s^ = 11 K also sets a new benchmark for solid‐state air‐stable SMMs.

## 4 Experimental Section

### General Procedures

The preparation of compounds **1**–**3** was carried out under rigorous anaerobic, anhydrous conditions under argon using standard Schlenk line and glove box techniques due to the instability of the reactants. All solvents were purified by standard methods and distilled prior to use. Dysprosium (Dy) metal, Yttrium (Y) metal, Iodine(I_2_), 18‐crown‐6, tert‐butanol, 1‐Adamantanol, and LiCH_3_ are commercially available and were used without further treatment. Powder X‐ray diffraction data (PXRD) were collected using a Bruker D8 advanced X‐ray diffractometer using Cu‐Kα radiation. Elements analysis was carried out by EA3000 Automatic Elemental Analyzer. IR spectra were collected on a Thermo Scientific Nicolet iS50 FTIR spectrometer. Raman spectra were performed on a Horiba Jobin‐Yvon LabRAM HR800 Evolution Laser Raman Spectrometer under 633 nm. Thermalgravimetric analyses were performed on a METTLER TOLEDO TGA/DSC3+ analyzer from room temperature to 800 °C with a constant rate of 10 °C min^−1^ in a flowing argon atmosphere. Accurate dysprosium/yttrium ratios of the magnetically diluted analogs were measured by using an Inductively Coupled Plasma Mass Spectrometer (ICP‐MS) NexION 350D instrument.

### Synthesis of [Dy(18‐C‐6)I_2_][I_3_] (1)

In an argon glove box, Dy Metal (81.3 mg, 0.5 mmol), 18‐crown‐6 (132.2 mg, 0.5 mmol), and I_2_ (317.3 mg 1.25 mmol) were mixed and added with 5 mL tetrahydrofuran (THF) solvent. The slurry was stirred for ≈4 days until Dy Metal completely dissolved and formed a cloudy solution, which was filtered through Celite. Deep yellow crystals of **1** suitable for X‐ray diffraction were isolated by layering 9 mL Et_2_O on the top of 3 mL solution at room temperature. Yield 415.5 mg, 78.3% (based on Dy). Anal. calcd for for C_24_H_48_Dy_2_I_10_O_12_: C 13.57, H 2.28; found: C 13.61, H 2.24. IR (KBr): *ν* = 1597(w), 1464(m), 1342(w), 1237(m), 1057(s), 958(s), 878(w), 821(s), 523 cm^−1^ (w).

### Synthesis of [Dy(18‐C‐6)(O^t^Bu)_2_][I_3_] (2)

To a solution of tert‐butanol (74.2 mg, 1 mmol) in THF (4 mL) was dropwise added with LiCH_3_ (1.6 mol L^−1^ in diethyl ether, 1 mmol) while stirring. After 2 h, the solution of **1** (0.5 mmol) in ≈5 mL THF was slowly added. A further stirring of 24 h was applied before filtering through Celite. Deep yellow crystals of **2** suitable for X‐ray diffraction were isolated by layering 9 mL Et_2_O on the top of 3 mL solution at room temperature. Yield 156.4 mg, 32.8% (based on Dy). Anal. calcd for for C_20_H_42_DyI_3_O_8_: C 25.05, H 4.38, found: C 25.20, H 4.44. IR (KBr): *ν* = 1468(s), 1453(w), 1368(w), 1349(s), 1283(w), 1246(w), 1233(w), 1218(w), 1203(s), 1097(s), 1006(s), 984(s), 847(w), 768(w), 549(w), 492 cm^−1^(w). **2@Y** was synthesized according to the same synthetic procedure for **2** except that the mixed Dy Metal (4.1 mg, 0.025 mmol) and Y Metal (42.2 mg, 0.475 mmol) were used in place of the pure Dy metal. The dysprosium/yttrium ratio is 8.15% measured by using an ICP‐MS NexION TM 350D instrument.

### Synthesis of [Dy(18‐C‐6)(1‐AdO)_2_][I_3_] (3)

To a solution of 1‐Adamantanol (152.2 mg, 1 mmol) in THF (4 mL) was dropwise added with LiCH_3_ (1.6 mol L^−1^ in diethyl ether, 1 mmol) under stirring. After 2 h, the solution of **1** (0.5 mmol) in ≈5 mL THF was slowly added. A further stirring of 24 h was applied before filtering through Celite. Deep yellow crystals of **3** suitable for X‐ray diffraction were isolated by layering 9 mL Et_2_O on the top of 3 mL solution at room temperature. Yield 157.6 mg, 28.4% (based on Dy). Anal. calcd for for C_32_H_54_DyI_3_O_8_: C 34.60, H 5.08, found: C 34.71, H 5.11. IR (KBr): *ν* = 1466(m), 1452(w), 1437(w), 1396(w), 1351(m), 1297(w), 1284(w), 1249(w), 1152(s), 1095(s), 996(m), 981(s), 951(m), 930(w), 843(m), 810(w), 778(w), 731(w), 635(m), 545(w), 471(w), 454(w), 415 cm^−1^ (w). **3@Y** was synthesized according to the same synthetic procedure for **3** was applied except that the mixed Dy Metal (4.1 mg, 0.025 mmol) and Y Metal (42.2 mg, 0.475 mmol) were used in place of the pure Dy metal. The dysprosium/yttrium ratio is 7.68% measured by using an ICP‐MS NexION TM 350D instrument. CCDC 2281966–2281969, 2281971–2281973, 2325500, and 2325501 contain the supplementary crystallographic data for this paper. These data can be obtained free of charge from The Cambridge Crystallographic Data Centre via www.ccdc.cam.ac.uk/data_request/cif.

### X‐Ray Crystallography Data

The diffraction data for **1**–**3** were collected on a Bruker SMART CCD diffractometer with Mo‐Kα radiation (*λ* = 0.71073 Å). The structures were solved by direct methods and were refined by full‐matrix least‐squares on all unique *F* ^2^ values, with anisotropic displacement parameters for all non‐hydrogen atoms. OLEX2 was employed for structure solution and refinement.^[^ ^41^ ^]^ To measure the steric hindrance of axial ligand in the complex, the percentage of buried volumes (%Vbur) was calculated by using the SambVca 2.1 tool.^[^ ^38^ ^]^ The XYZ files involved in all calculations wee extracted from their single‐crystal XRD data. The parameters were set as follows: sphere radius 6 Å; mesh spacing 0.10; bond radii scaled by 1.17; H atoms wee included.

### Magnetic Property Measurements

The magnetic data were recorded on Quantum Design MPMS‐XL7 SQUID and MPMS‐squid VSM‐094 magnetometer. The direct current (DC) magnetic susceptibility and magnetization data were collected in cooling mode from 300 to 2 K with an external magnetic field of 1000 Oe. The magnetization data were collected at 2 K with variable applied fields from 0 to 5 T. Alternating current (ac) magnetic susceptibility measurements have been performed at frequencies of between 1 and 1218 Hz with an oscillating field of 3.5 Oe. Magnetic relaxation times, τ, were extracted from a simultaneous fit of in‐phase (*χ'*) and out‐of‐phase (*χ''*) components of the magnetic susceptibility to a generalized Debye model. The α values extracted from fits to *χ'* and *χ''* data were used to calculate uncertainty ranges for τ according to the equation $\tau_{\pm }=\;exp\left\{\ln\tau \pm \sqrt{\frac{\pi^{2}}{6}\left(\frac{1}{{\left(1-\alpha \right)}^{2}}-1\right)}\right\}$.^[^ ^42^, ^43^ ^]^ The DC decay measurements were collected by magnetizing the sample using a field of 50 kOe, and then returning the field to 0 Oe and measuring the magnetization as a function of time. The crushed crystalline samples were embedded in eicosane to avoid any field‐induced crystal reorientation. A diamagnetic correction has been calculated from Pascal constants and embedding eicosane has been applied to the observed magnetic susceptibility.

### Ab Initio Calculation

Ab initio calculations at the SA‐CASSCF/RASSI level were performed using the OpenMolcas program.^[^ ^39^ ^]^ The cationic coordinates in **2** and **3** were directly extracted from their single crystal structures without optimizations. The basis sets were chosen from the MOLCAS ANO‐RCC library^[^ ^44^ ^]^: VTZP quality for Dy atoms, VDZP for the O atoms, and VDZ for the rest of the atoms. All 21 sextets, 224 quartets, and 490 doublets were considered in the state‐averaged calculations. Then 21 sextets, 128 quartets, and 130 doublets were chosen to construct and diagonalize the spin–orbit (SO) coupling Hamiltonian in the RASSI module.^[^ ^45^ ^]^ The Cholesky decomposition for two‐election integrals was employed in whole calculations to ensure the calculation accuracy and save disk space.

## Conflict of Interest

The authors declare no conflict of interest.

## Supporting information

Supporting Information

## Data Availability

The data that support the findings of this study are available from the corresponding author upon reasonable request.
