# Supplementary material for: Bis‐Alkoxide Dysprosium(III) Crown Ether Complexes Exhibit Tunable Air Stability and Record Energy Barrier
Source: Adv Sci (Weinh). 2024 Feb 23;11(17):2308548. doi: 10.1002/advs.202308548 (PMC11077650; doi:10.1002/advs.202308548)
Supplement: Supplementary file 1 — Supporting Information [file ADVS-11-2308548-s001.pdf]

ADVANCED SCIENCE
Open Access

Supporting Information

for *Adv. Sci.*, DOI 10.1002/advs.202308548

Bis-Alkoxide Dysprosium(III) Crown Ether Complexes Exhibit Tunable Air Stability and Record Energy Barrier

*Wen-Jie Xu, Qian-Cheng Luo, Zi-Han Li, Yuan-Qi Zhai and Yan-Zhen Zheng**

Supporting Information

Bis-Alkoxide Dysprosium(III) Crown Ether Complexes Exhibit Tunable Air Stability and Record Energy Barrier

*Wen-Jie Xu, Qian-Cheng Luo, Zi-Han Li, Yuan-Qi Zhai, and Yan-Zhen Zheng**

Contents

1. Raman and Infrared Spectra

2. X-ray Crystallography Data

3. Magnetic Property Measurements

4. *Ab initio* Calculations

5. References

1. Raman and Infrared Spectra

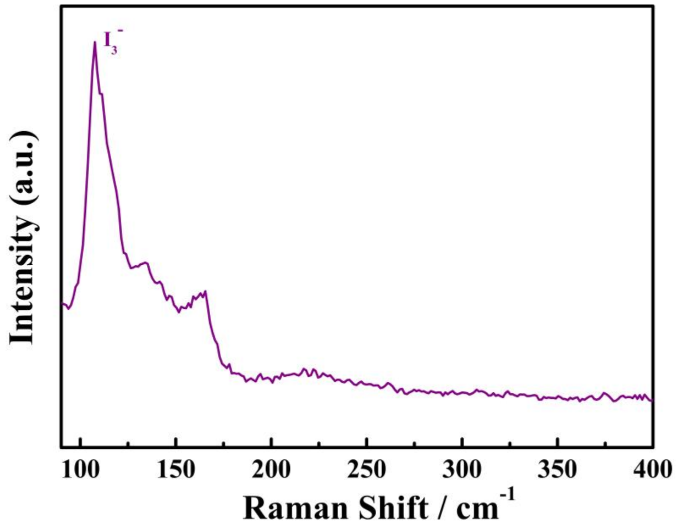

Figure S1. Raman spectrum for **1**.

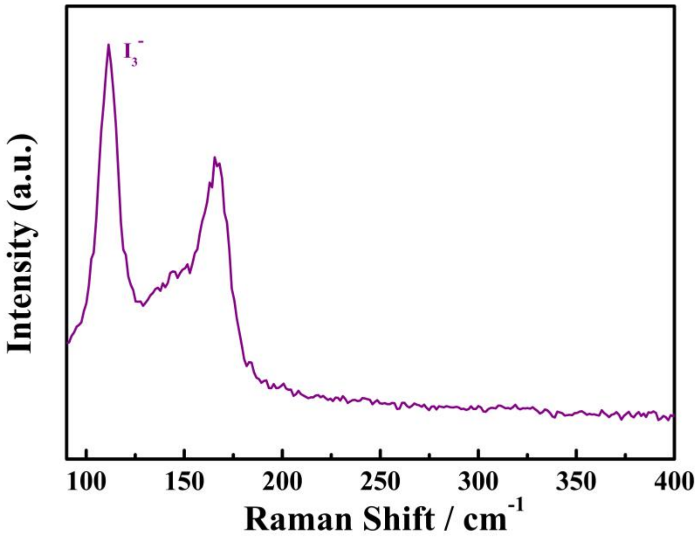

Figure S2. Raman spectrum for **2**.

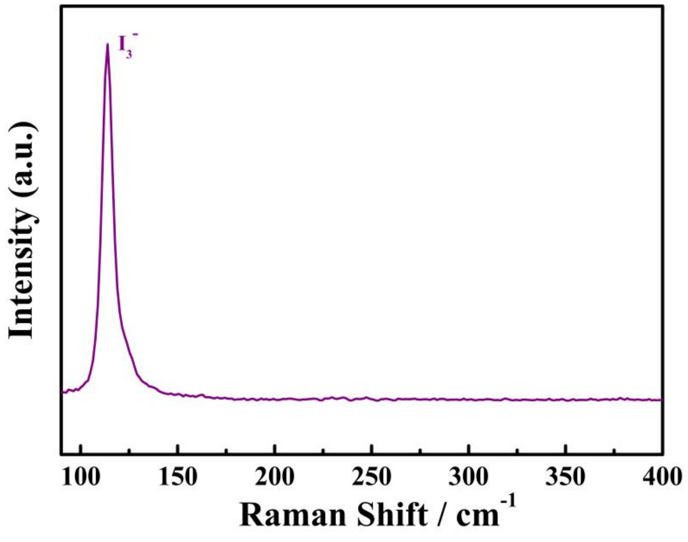

Figure S3. Raman spectrum for **3**.

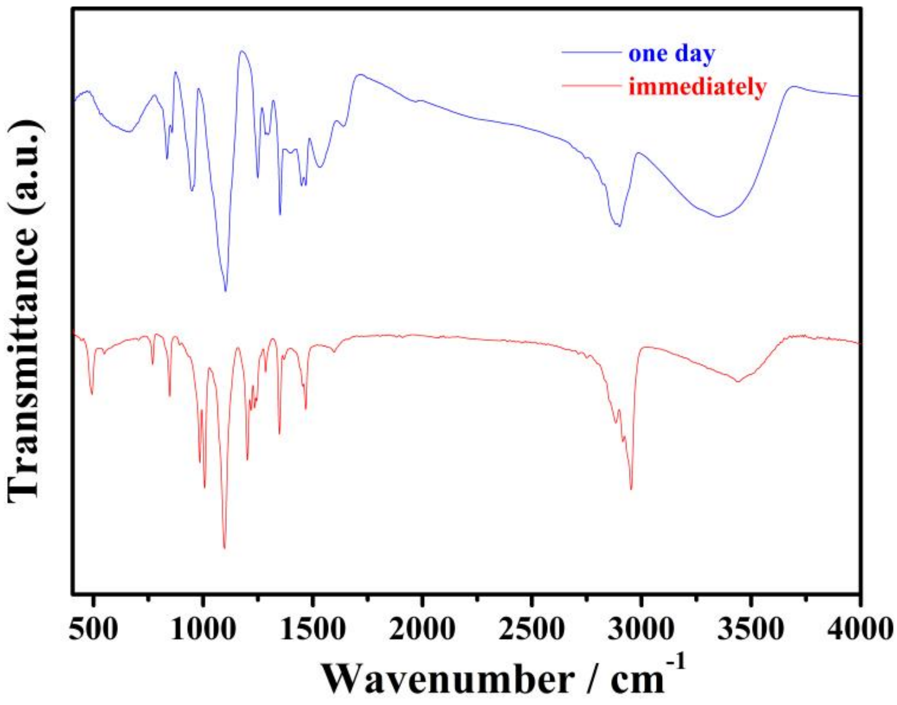

Figure S4. Infrared spectrum of **2** over time in air atmosphere.

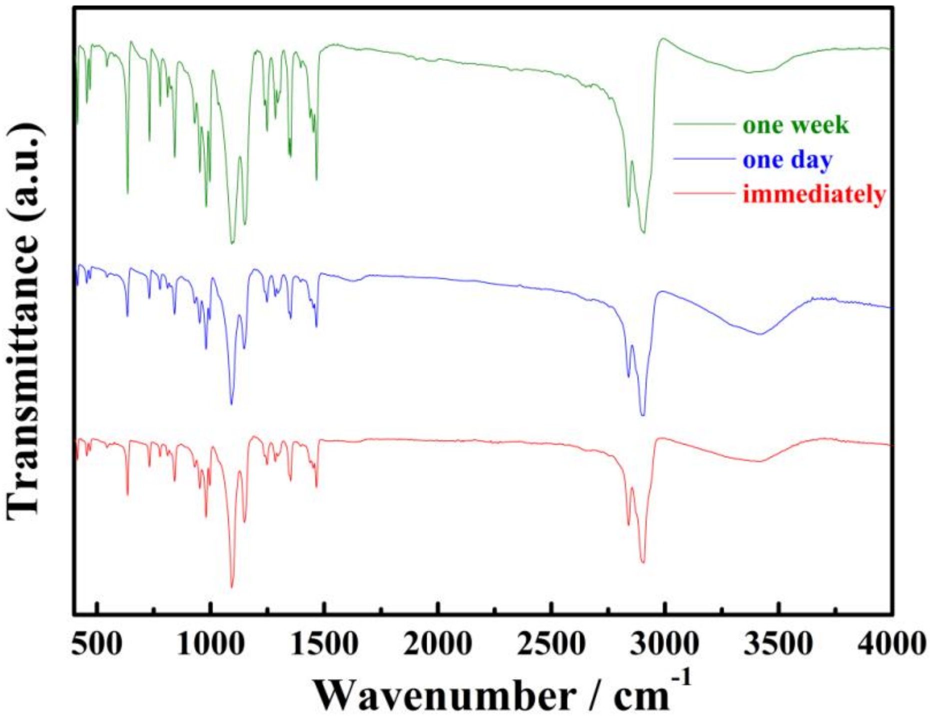

Figure S5. Infrared spectrum of **3** over time in air atmosphere.

2. X-ray Crystallography Data

Table S1. Crystal data and structure refinement for **1**, **2**, and **2Y**.

**Compound reference**	**1**(298 K)	**2**(298 K)	**2**(150 K)	**2**(100 K)	**2Y**(150 K)
Chemical formula	$C_{24}H_{48}Dy_2I_{10}O_{12}$	$C_{20}H_{42}DyI_3O_8$	$C_{20}H_{42}DyI_3O_8$	$C_{20}H_{42}DyI_3O_8$	$C_{20}H_{42}YI_3O_8$
Formula Mass	2122.62	953.73	953.73	953.73	880.14
Temperature (K)	298	298	150	100	150
Crystal system	orthorhombic	monoclinic	monoclinic	monoclinic	monoclinic
Space group	$Pna2_1$	$P2_1/n$	$P2_1/n$	$P2_1/n$	$P2_1/n$
a (Å)	16.736(3)	9.7441(12)	9.6138(17)	9.592(3)	9.5532(4)
b (Å)	10.4399(19)	14.0048(17)	13.906(2)	13.924(4)	13.8504(7)
c (Å)	29.159(5)	24.153(3)	23.875(4)	23.912(7)	23.7590(10)
α (°)	90	90	90	90	90
β (°)	90	92.167(2)	92.513(2)	92.580(4)	92.633(2)
γ (°)	90	90	90	90	90
Unit cell volume (Å^3)	5094.7(16)	3293.7(7)	3188.8(10)	3190.2(16)	3140.4(2)
Z	4	4	4	4	4
ρ_{calc} (g/cm^3)	2.767	1.923	1.987	1.986	1.862
Absorption coefficient, μ(mm^{-1})	9.013	5.114	5.282	5.279	4.841
F(000)	3800.0	1804.0	1804.0	1804.0	1696.0
Reflections collected	43064	37113	26912	24932	69360
Independent reflections	12389	7607	7316	7429	7216
R_{int}	0.0341	0.0299	0.0286	0.0598	0.0741
GOF on F^2	1.011	1.035	1.033	1.064	0.757
R_1 (I>2σ/all data)	0.0365/0.0487	0.0462/0.0678	0.0242/0.0329	0.0392/0.0545	0.0274/0.0379
wR_2 (I>2σ/all data)	0.0813/0.0868	0.1192/0.1318	0.0526/0.0555	0.0893/0.0941	0.0895/0.1014

Table S2. Crystal data and structure refinement for **3** and **3Y**.

**Compound reference**	**3**(298 K)	**3**(150 K)	**3**(100 K)	**3Y**(150 K)
Chemical formula	$C_{32}H_{54}DyI_3O_8$	$C_{32}H_{54}DyI_3O_8$	$C_{32}H_{54}DyI_3O_8$	$C_{32}H_{54}YI_3O_8$
Formula Mass	1109.95	1109.95	1109.95	1036.36
Temperature (K)	298	150	100	100
Crystal system	orthorhombic	orthorhombic	orthorhombic	orthorhombic
Space group	$Cmc2_1$	$Cmc2_1$	$Cmc2_1$	$Cmc2_1$
a (Å)	10.8340(15)	10.811(5)	10.7924(18)	10.7586(9)
b (Å)	18.962(3)	18.727(11)	18.608(3)	18.5546(9)
c (Å)	19.025(3)	19.003(8)	18.912(3)	18.8722(11)
α (°)	90	90	90	90
β (°)	90	90	90	90
γ (°)	90	90	90	90
Unit cell volume (Å^3)	3908.3(9)	3847(3)	3798.0(11)	3767.3(4)
Z	4	4	4	4
ρ_{calc} (g/cm^3)	1.886	1.916	1.941	1.827
Absorption coefficient, μ(mm^{-1})	4.324	4.393	4.450	4.051
F(000)	2140.0	2140.0	2140.0	2032.0
Reflections collected	15620	21292	16196	50350
Independent reflections	4265	4531	4265	4569
R_{int}	0.0206	0.0283	0.0218	0.0809
GOF on F^2	1.037	1.042	1.061	1.061
R_1 (I>2σ/all data)	0.0230/0.0253	0.0193/0.0204	0.0179/0.0186	0.0343/0.0424
wR_2 (I>2σ/all data)	0.0535/0.0545	0.0472/0.0477	0.0427/0.0429	0.0760/0.0832

Table S3. Selected bond lengths (Å) and angles (°) for **1**(298 K).

Dy1–I1	3.024(2)	Dy1–I2	3.002(2)
Dy1–O1	2.438(8)	Dy1–O2	2.450(8)
Dy1–O3	2.432(10)	Dy1–O4	2.452(9)
Dy1–O5	2.476(9)	Dy1–O6	2.463(10)
I1–Dy1–I2	165.84(3)		
I1–Dy1–O1	82.9(2)	I2–Dy1–O1	85.3(2)
I1–Dy1–O2	84.4(2)	I2–Dy1–O2	83.4(2)
I1–Dy1–O3	96.6(3)	I2–Dy1–O3	84.2(3)
I1–Dy1–O4	76.9(3)	I2–Dy1–O4	115.5(3)
I1–Dy1–O5	113.1(3)	I2–Dy1–O5	79.8(3)
I1–Dy1–O6	85.9(3)	I2–Dy1–O6	95.7(3)
O1–Dy1–O2	63.6(3)	O2–Dy1–O3	63.2(4)
O3–Dy1–O4	62.2(3)	O4–Dy1–O5	63.2(3)
O5–Dy1–O6	62.7(3)	O6–Dy1–O1	63.4(3)

Table S4. Selected bond lengths (Å) and angles (°) for **2**(298 K).

Dy1–O1A	2.067(5)	Dy1–O2A	2.069(5)
Dy1–O1E	2.608(5)	Dy1–O4E	2.629(5)
Dy1–O2E	2.627(5)	Dy1–O5E	2.593(5)
Dy1–O3E	2.635(5)	Dy1–O6E	2.585(5)
O1A–Dy–O2A	178.04(19)		
O1A–Dy–O1E	94.1(2)	O2A–Dy–O1E	87.8(2)
O1A–Dy–O2E	90.07(19)	O2A–Dy–O2E	90.95(19)
O1A–Dy–O3E	87.6(2)	O2A–Dy–O3E	91.4(2)
O1A–Dy–O4E	91.43(19)	O2A–Dy–O4E	86.61(19)
O1A–Dy–O5E	87.30(18)	O2A–Dy–O5E	91.68(18)
O1A–Dy–O6E	88.5(2)	O2A–Dy–O6E	92.5(2)
O1E–Dy–O2E	59.27(19)	O2E–Dy–O3E	59.26(19)
O3E–Dy–O4E	59.99(19)	O4E–Dy–O5E	60.92(18)
O5E–Dy–O6E	60.88(19)	O6E–Dy–O1E	60.20(19)

Table S5. Selected bond lengths (Å) and angles (°) for **2**(150 K).

Dy1–O1A	2.066(2)	Dy1–O2A	2.069(2)
Dy1–O1E	2.656(2)	Dy1–O4E	2.605(2)
Dy1–O2E	2.606(2)	Dy1–O5E	2.623(2)
Dy1–O3E	2.598(2)	Dy1–O6E	2.659(2)
O1A–Dy–O2A	177.51(10)		
O1A–Dy–O1E	85.60(9)	O2A–Dy–O1E	92.07(9)
O1A–Dy–O2E	91.42(8)	O2A–Dy–O2E	86.66(9)
O1A–Dy–O3E	91.72(9)	O2A–Dy–O3E	88.75(9)
O1A–Dy–O4E	87.55(9)	O2A–Dy–O4E	94.80(9)
O1A–Dy–O5E	91.90(9)	O2A–Dy–O5E	89.99(9)
O1A–Dy–O6E	92.31(9)	O2A–Dy–O6E	87.25(9)
O1E–Dy–O2E	60.54(7)	O2E–Dy–O3E	61.23(8)
O3E–Dy–O4E	60.21(8)	O4E–Dy–O5E	59.90(8)
O5E–Dy–O6E	59.33(8)	O6E–Dy–O1E	59.56(8)

Table S6. Selected bond lengths (Å) and angles (°) for **2**(100 K).

Dy1–O1A	2.070(4)	Dy1–O2A	2.067(4)
Dy1–O1E	2.602(4)	Dy1–O4E	2.660(4)
Dy1–O2E	2.631(4)	Dy1–O5E	2.610(4)
Dy1–O3E	2.663(3)	Dy1–O6E	2.601(3)
O1A–Dy–O2A	177.53(15)		
O1A–Dy–O1E	94.56(14)	O2A–Dy–O1E	87.81(14)
O1A–Dy–O2E	89.58(14)	O2A–Dy–O2E	92.22(14)
O1A–Dy–O3E	87.01(13)	O2A–Dy–O3E	92.44(13)
O1A–Dy–O4E	92.38(13)	O2A–Dy–O4E	85.27(14)
O1A–Dy–O5E	87.10(13)	O2A–Dy–O5E	91.08(13)
O1A–Dy–O6E	88.95(13)	O2A–Dy–O6E	91.62(14)
O1E–Dy–O2E	59.80(11)	O2E–Dy–O3E	59.31(12)
O3E–Dy–O4E	59.85(12)	O4E–Dy–O5E	60.40(11)
O5E–Dy–O6E	61.06(11)	O6E–Dy–O1E	60.37(12)

Table S7. Selected bond lengths (Å) and angles (°) for **3**(298 K).

Dy1–O1A	2.058(6)	Dy1–O2A	2.049(6)
Dy1–O1E	2.656(7)	Dy1–O4E	2.650(7)
Dy1–O2E	2.630(4)	Dy1–O3E^{1}	2.625(4)
Dy1–O3E	2.625(4)	Dy1–O2E^{1}	2.630(4)
O1A–Dy–O2A	178.0(3)		
O1A–Dy–O1E	86.7(2)	O2A–Dy–O1E	91.3(2)
O1A–Dy–O2E	90.95(14)	O2A–Dy–O2E	88.03(15)
O1A–Dy–O3E	90.53(14)	O2A–Dy–O3E	90.49(15)
O1A–Dy–O4E	88.7(2)	O2A–Dy–O4E	93.3(2)
O1A–Dy–O3E^{1}	90.53(14)	O2A–Dy–O3E^{1}	90.49(15)
O1A–Dy–O2E^{1}	90.95(14)	O2A–Dy–O2E^{1}	88.03(15)
O1E–Dy–O2E	60.00(11)	O2E–Dy–O3E	60.42(14)
O3E–Dy–O4E	59.72(11)	O4E–Dy–O3E^{1}	59.72(11)
O3E^{1}–Dy–O2E^{1}	60.42(14)	O2E^{1}–Dy–O1E	60.00(11)

Table S8. Selected bond lengths (Å) and angles (°) for **3**(150 K).

Dy1–O1A	2.059(5)	Dy1–O2A	2.061(5)
Dy1–O1E	2.673(5)	Dy1–O4E	2.677(5)
Dy1–O2E	2.626(3)	Dy1–O3E^{1}	2.635(3)
Dy1–O3E	2.635(3)	Dy1–O2E^{1}	2.626(3)
O1A–Dy–O2A	177.8(2)		
O1A–Dy–O1E	93.38(19)	O2A–Dy–O1E	88.82(17)
O1A–Dy–O2E	90.39(12)	O2A–Dy–O2E	90.73(11)
O1A–Dy–O3E	87.87(11)	O2A–Dy–O3E	91.02(11)
O1A–Dy–O4E	91.70(19)	O2A–Dy–O4E	86.10(18)
O1A–Dy–O3E^{1}	87.87(11)	O2A–Dy–O3E^{1}	91.02(11)
O1A–Dy–O2E^{1}	90.39(12)	O2A–Dy–O2E^{1}	90.73(11)
O1E–Dy–O2E	59.55(8)	O2E–Dy–O3E	60.76(11)
O3E–Dy–O4E	59.87(8)	O4E–Dy–O3E^{1}	59.87(8)
O3E^{1}–Dy–O2E^{1}	60.76(11)	O2E^{1}–Dy–O1E	59.55(8)

Table S9. Selected bond lengths (Å) and angles (°) for **3**(100 K).

Dy1–O1A	2.057(4)	Dy1–O2A	2.056(4)
Dy1–O1E	2.667(5)	Dy1–O4E	2.676(5)
Dy1–O2E	2.619(3)	Dy1–O3E^1	2.633(3)
Dy1–O3E	2.633(3)	Dy1–O2E^1	2.619(3)
O1A–Dy–O2A	177.49(19)		
O1A–Dy–O1E	94.05(16)	O2A–Dy–O1E	88.47(15)
O1A–Dy–O2E	90.64(10)	O2A–Dy–O2E	90.64(10)
O1A–Dy–O3E	87.70(10)	O2A–Dy–O3E	91.04(10)
O1A–Dy–O4E	91.35(16)	O2A–Dy–O4E	86.14(16)
O1A–Dy–O3E^1	87.70(10)	O2A–Dy–O3E^1	91.04(10)
O1A–Dy–O2E^1	90.64(10)	O2A–Dy–O2E^1	90.64(10)
O1E–Dy–O2E	59.54(7)	O2E–Dy–O3E	60.79(10)
O3E–Dy–O4E	59.86(7)	O4E–Dy–O3E^1	59.86(7)
O3E^1–Dy–O2E^1	60.79(10)	O2E^1–Dy–O1E	59.54(7)

Table S10. Continuous Shape Measures (CShM) calculations for the potential coordination geometries of **1-3**. The lowest CShMs value is highlighted.

**Symmetry** / **Structure**	OP-8 (D_{8h})	HPY-8 (C_{7v})	**HBPY-8 (D_{6h})**	CU-8 (O_h)	SAPR-8 (D_{4d})	TDD-8 (D_{2d})	JGBF-8 (D_{2d})	JETBPY-8 (D_{3h})
**1**(298 K)	35.285	23.915	**4.087**	5.768	8.060	6.990	10.018	27.492
**2**(298 K)	30.677	21.147	**1.019**	9.629	18.428	15.778	8.928	24.978
**2**(150 K)	30.759	21.256	**1.100**	9.536	18.315	15.800	8.642	24.698
**2**(100 K)	30.859	21.391	**1.108**	9.520	18.346	15.797	8.664	24.677
**3**(298 K)	30.752	21.427	**1.077**	9.430	18.917	16.161	9.430	25.206
**3**(150 K)	30.663	21.502	**1.100**	9.394	18.820	16.062	9.271	25.238
**3**(100 K)	30.667	21.410	**1.108**	9.421	18.849	16.090	9.227	25.170

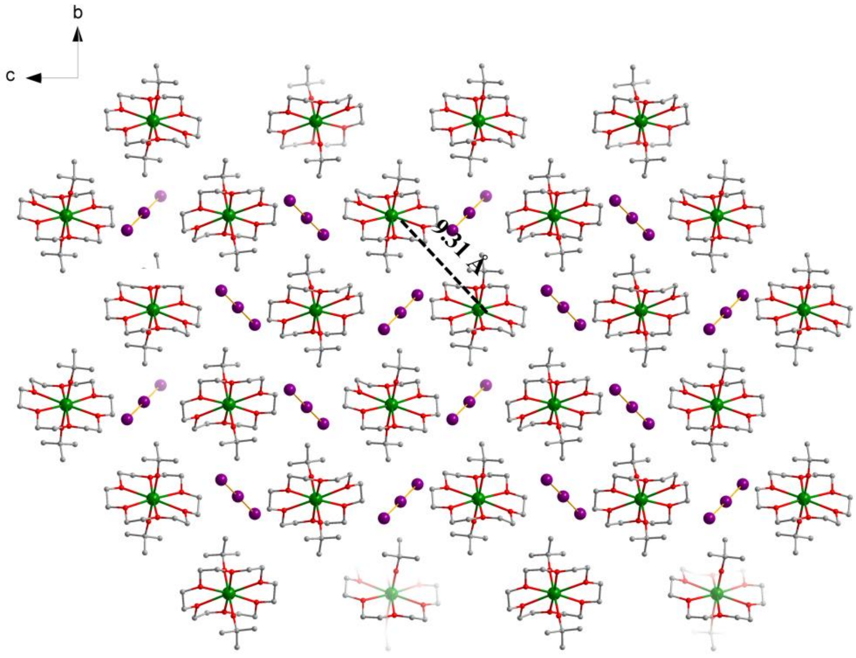

Figure S6. The packing diagram for **2**(298 K) shown along the *a* axis gives the shortest intermolecular Dy···Dy distance of 9.31 Å (9.19 Å for **2**(100 K)). Hydrogen atoms are omitted for clarity.

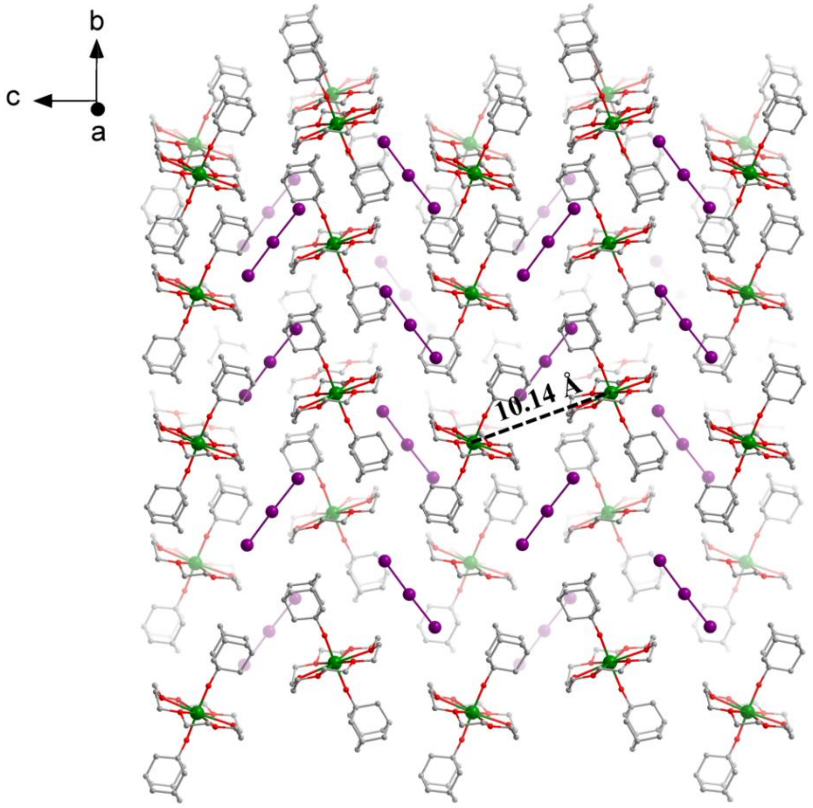

Figure S7. The packing diagram for **3**(298 K) shown along the *a* axis gives the shortest intermolecular Dy···Dy distance of 10.14 Å (10.06 Å for **3**(100 K)). Hydrogen atoms are omitted for clarity.

Table S11. Fractional atomic coordinates (×10[4]) and equivalent isotropic temperature factors (U_{eq}, Å^2×10^3) for **2**(298 K).

Atom	x	y	z	U_{eq}
Dy1	3883.4(3)	2488.7(2)	3663.6(2)	**44.04(10)**
O1A	2697(5)	3682(3)	3803(2)	**60.4(11)**
O2A	5013(5)	1266(4)	3527(2)	**64.2(12)**
C1A	1871(8)	4460(5)	3911(3)	**63.8(14)**
C11A	2724(13)	5337(7)	3916(6)	**133(5)**
C12A	829(13)	4572(9)	3453(6)	**138(5)**
C13A	1207(10)	4357(8)	4452(4)	**94(2)**
C2A	5765(10)	428(7)	3484(4)	**86.8(15)**
C21A	6519(12)	479(8)	2965(5)	**111(2)**
C22A	6837(12)	394(8)	3938(5)	**106(2)**
C23A	4873(12)	-405(8)	3561(5)	**110(2)**
O1E	6122(6)	3468(5)	3528(3)	**85.2(17)**
O2E	4467(6)	3156(4)	2683(2)	**80.5(16)**
O3E	2259(6)	2137(4)	2790(2)	**80.6(16)**
O4E	1750(6)	1357(4)	3752(2)	**76.5(15)**
O5E	3236(6)	1898(4)	4637(2)	**72.1(14)**
O6E	5421(6)	2962(5)	4515(2)	**78.9(16)**
C1E	2316(10)	1133(7)	4689(4)	**92(3)**
C2E	4210(11)	1976(8)	5088(3)	**92(3)**
C3E	4858(12)	2915(9)	5050(4)	**97(3)**
C4E	6400(12)	3719(9)	4469(5)	**119(4)**
C5E	7138(10)	3576(10)	3970(4)	**121(4)**
C6E	5421(6)	2962(5)	4515(2)	**78.9(16)**
C7E	5583(11)	3787(8)	2611(4)	**110(4)**
C8E	3361(9)	3312(7)	2288(4)	**101(3)**
C9E	2641(13)	2412(7)	2242(4)	**100(3)**
C10E	1475(13)	1275(8)	2790(4)	**122(4)**
C11E	779(10)	1223(9)	3312(4)	**108(4)**
C12E	1183(10)	1239(8)	4291(4)	**100(3)**
I1	9042.4(6)	7362.2(4)	3942.2(2)	**69.84(16)**
I2	11442.6(8)	8283.3(6)	4462.8(4)	**109.0(3)**
I3	6611.8(8)	6480.2(7)	3432.2(4)	**125.3(3)**

Table S12. Fractional atomic coordinates (×10[4]) and equivalent isotropic temperature factors (U_{eq}, Å^2×10^3) for **3**(298 K).

Atom	x	y	z	U_{eq}
Dy1	5000	4075.6(2)	5695.2(2)	**35.18(9)**
O1A	5000	5079(3)	6109(3)	**46.4(14)**
O2A	5000	3063(3)	5319(3)	**49.9(15)**
C1A	5000	5079(3)	6109(3)	**46.4(14)**
C11A	5000	5601(5)	7262(5)	**56(2)**
C12A	6146(7)	6156(4)	6278(4)	**67(2)**
C13A	5000	6295(5)	7677(5)	**52(2)**
C14A	6148(8)	6708(5)	7477(4)	**69(2)**
C15A	6152(9)	6858(4)	6692(4)	**75(3)**
C16A	5000	7283(5)	6516(6)	**84(4)**
C2A	5000	2392(4)	5004(4)	**37.2(17)**
C21A	5000	2458(5)	4220(6)	**74(3)**
C22A	3875(8)	1968(4)	5220(5)	**80(3)**
C23A	5000	1730(5)	3856(5)	**73(4)**
C24A	3865(9)	1332(5)	4077(5)	**83(3)**
C25A	3872(9)	1232(4)	4860(6)	**88(3)**
C26A	3865(9)	1332(5)	4077(5)	**83(3)**
C1E	6091(8)	3286(5)	7274(5)	**76(2)**
O1E	5000	3616(4)	7014(3)	**55.2(16)**
O2E	7099(4)	3788(3)	6327(3)	**56.3(11)**
O3E	7092(4)	4331(3)	5047(3)	**52.8(10)**
O4E	5000	4639(3)	4421(3)	**51.2(15)**
C2E	7138(7)	3738(5)	7070(4)	**72(2)**
C3E	7092(4)	4331(3)	5047(3)	**52.8(10)**
C4E	8134(9)	3954(6)	5267(6)	**78(3)**
C5E	7104(7)	4478(4)	4314(4)	**66(2)**
C6E	6102(7)	4979(5)	4185(4)	**68(2)**
I1	10000	2236.8(4)	4324.9(4)	**73.1(2)**
I2	10000	3458.2(3)	3387.7(3)	**51.83(15)**
I3	10000	2236.8(4)	4324.9(4)	**73.1(2)**

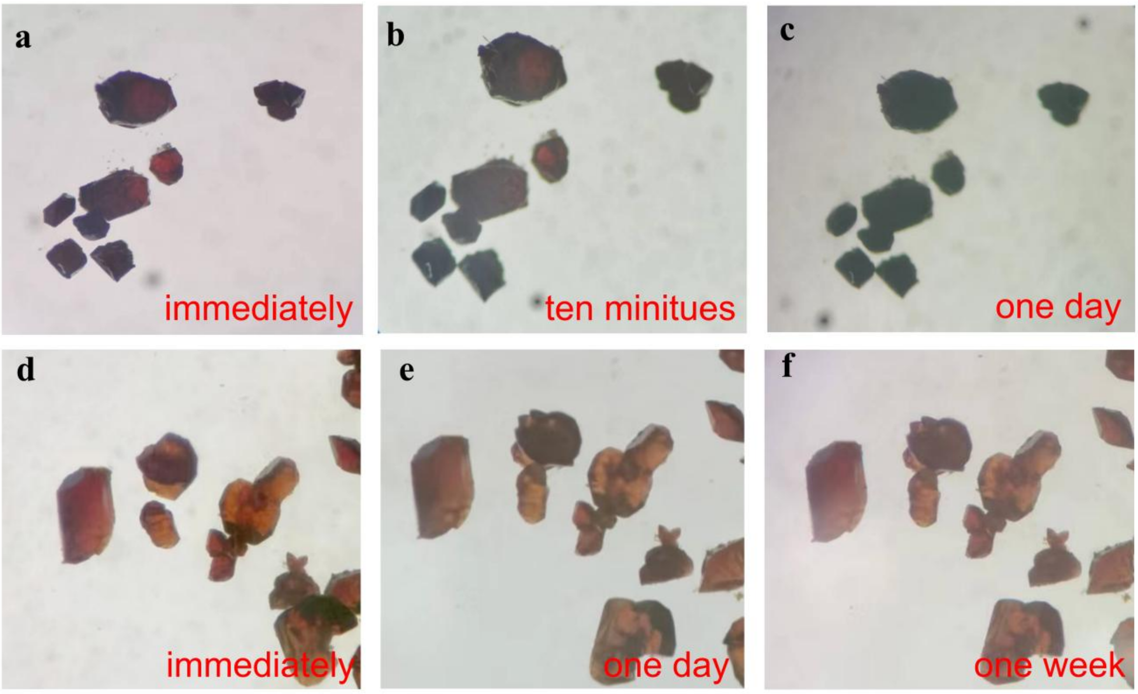

Figure S8. Photographs of single-crystal samples of **2**(a-c) and **3**(d-f) exposed to air over time.

3. Magnetic Property Measurements

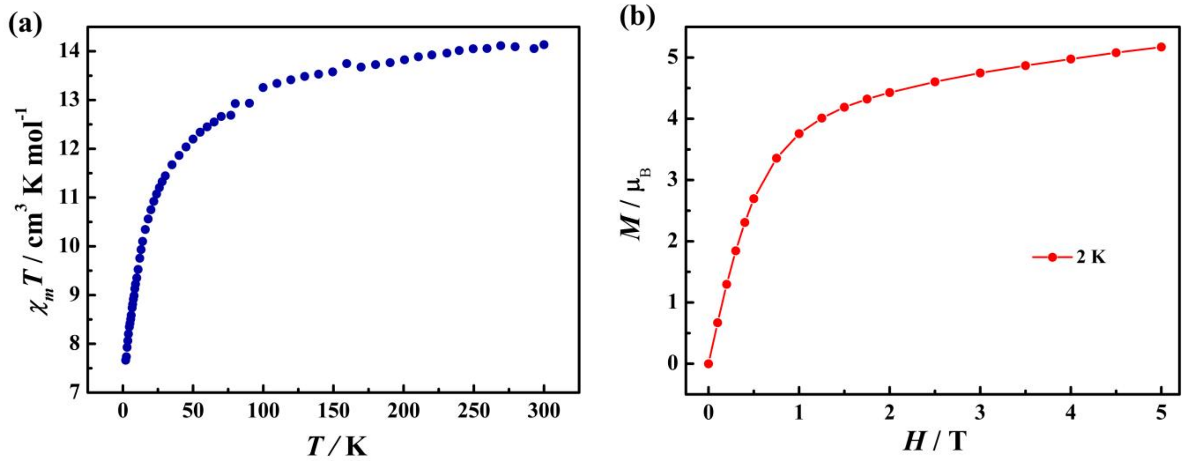

Figure S9. (a) Temperature-dependence of the DC magnetic susceptibility of **1** under an applied DC field of 1000 Oe. (b) Field-dependence of the magnetization of **1** at 2 K. The solid line is guide for the eyes.

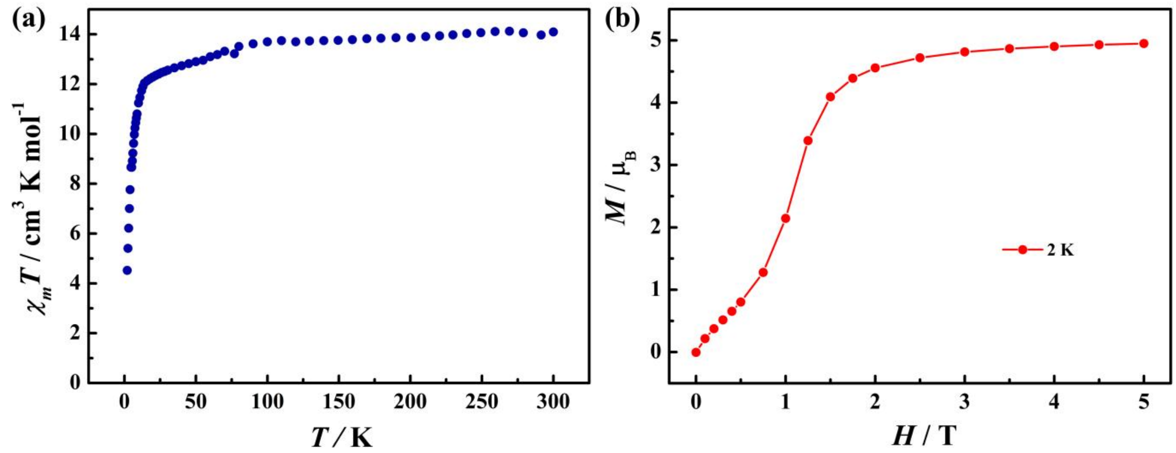

Figure S10. (a) Temperature-dependence of the DC magnetic susceptibility of **2** under an applied DC field of 1000 Oe. (b) Field-dependence of the magnetization of **2** at 2 K. The solid line is guide for the eyes.

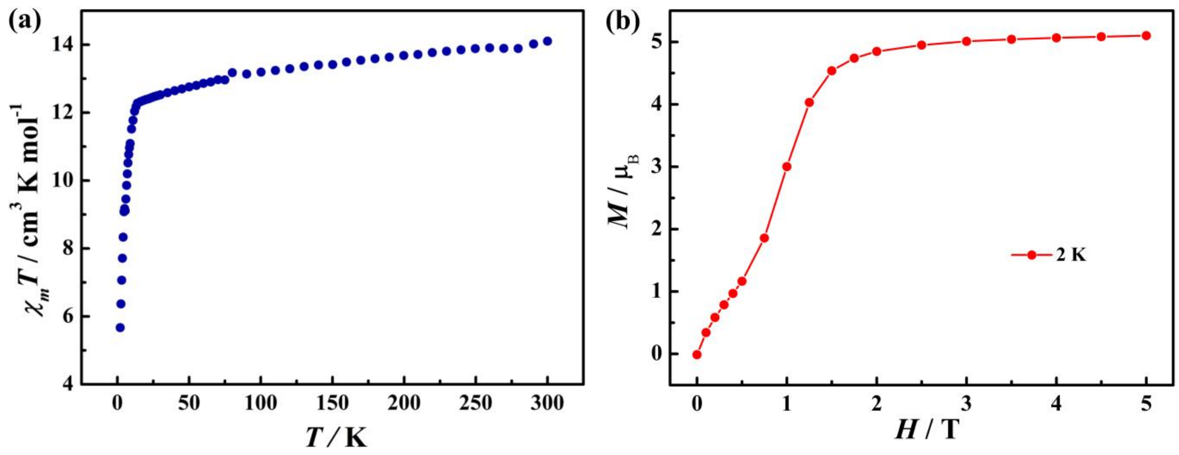

Figure S11. (a) Temperature-dependence of the DC magnetic susceptibility of **3** under an applied DC field of 1000 Oe. (b) Field-dependence of the magnetization of **3** at 2 K. The solid line is guide for the eyes.

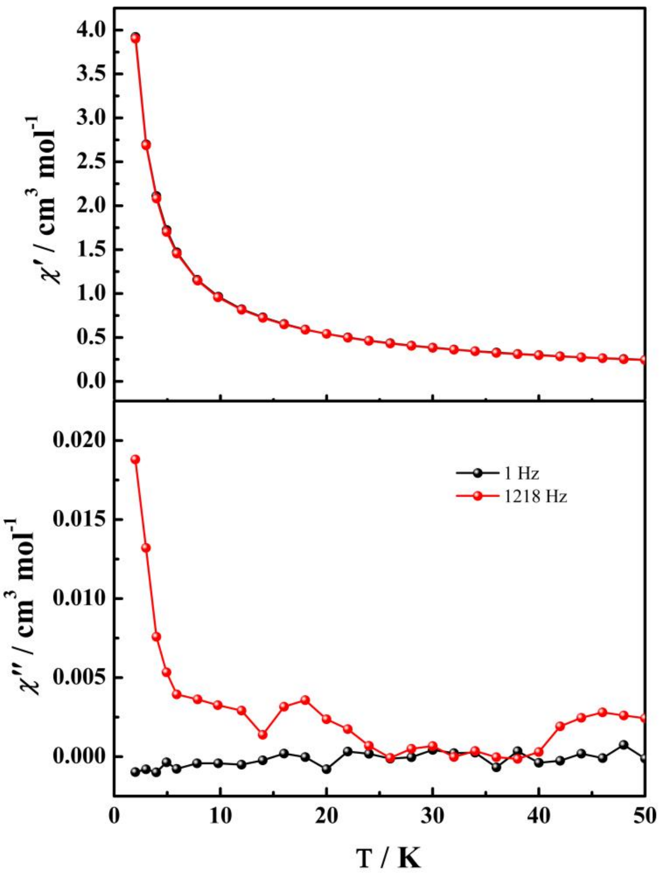

Figure S12. Temperature dependence of the in-phase (χ', top) and out-of-phase (χ'', bottom) ac susceptibility for **1** in a zero DC field with an AC frequency of 1 and 1218 Hz.

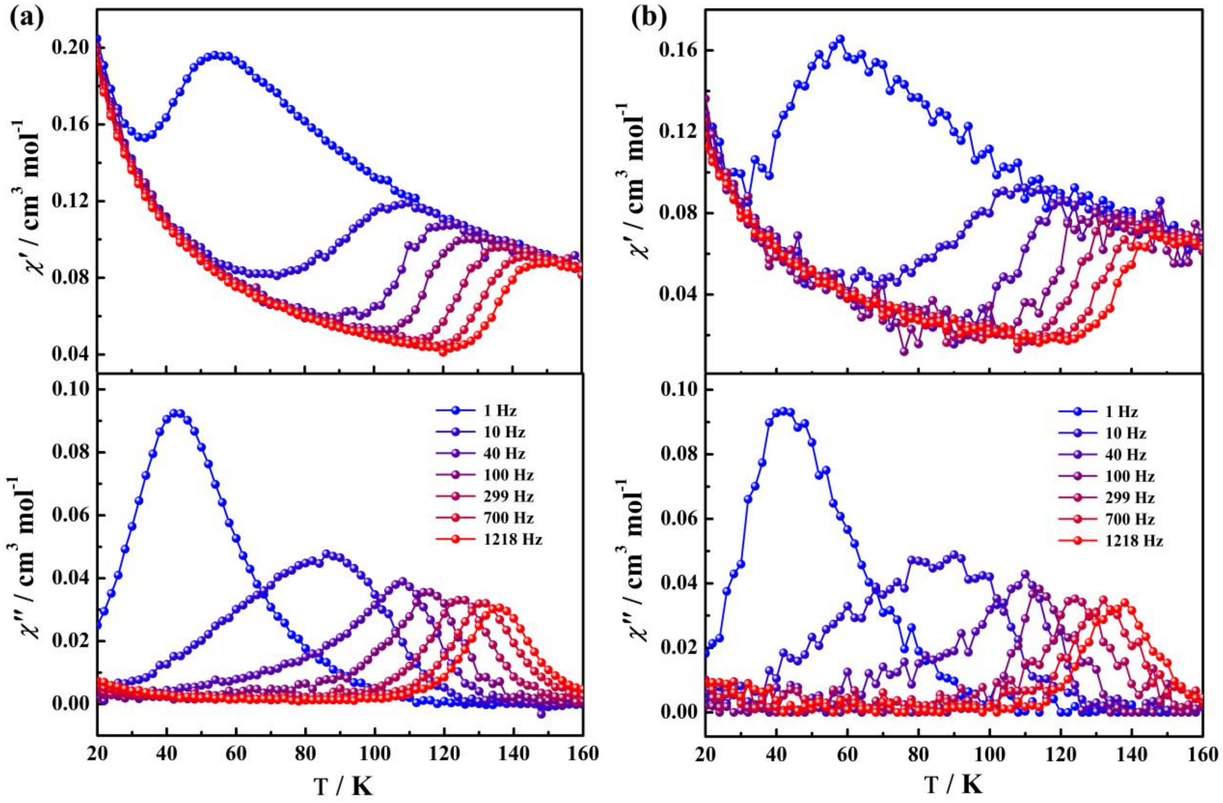

Figure S13. Temperature dependence of the in-phase (χ', top) and out-of-phase (χ'', bottom) AC susceptibility for **2** (a) and **2@Y** (b) in a zero DC field with an AC frequency of 1–1218 Hz.

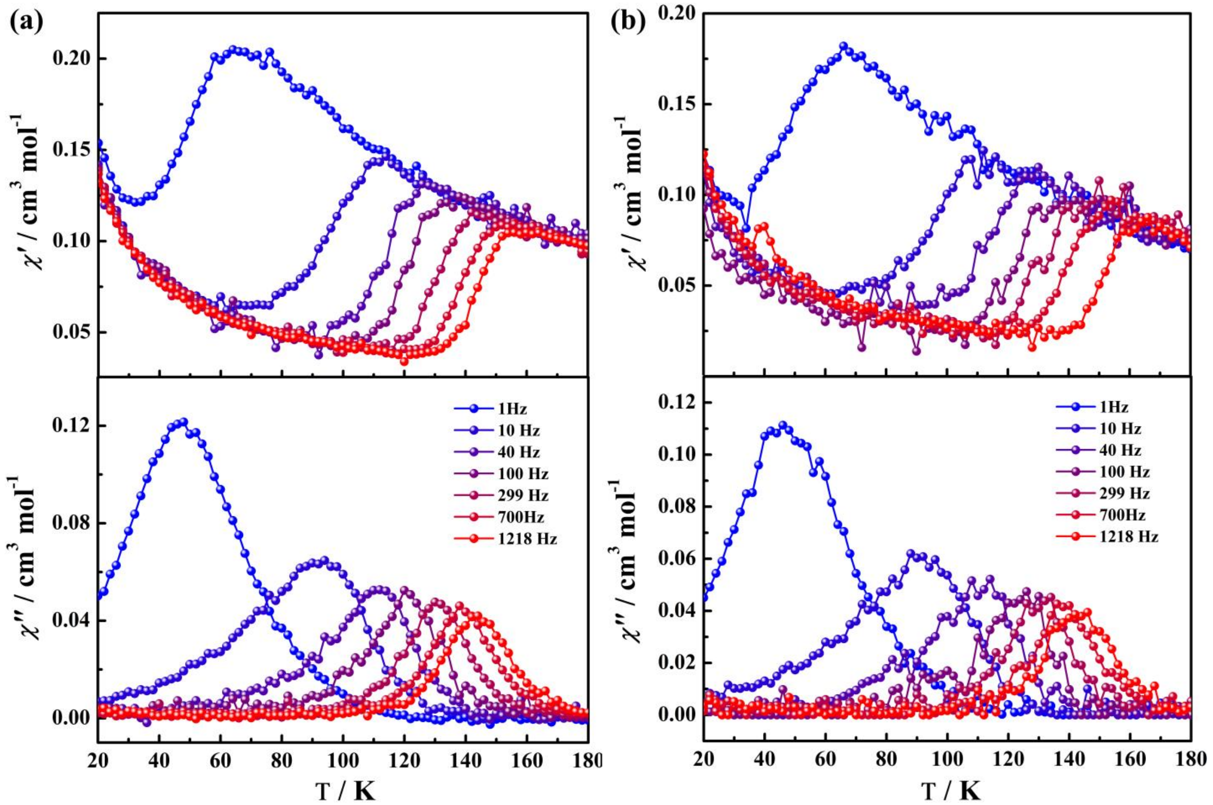

Figure S14. Temperature dependence of the in-phase (χ', top) and out-of-phase (χ'', bottom) AC susceptibility for **3** (a) and **3@Y** (b) in a zero DC field with an AC frequency of 1–1218 Hz.

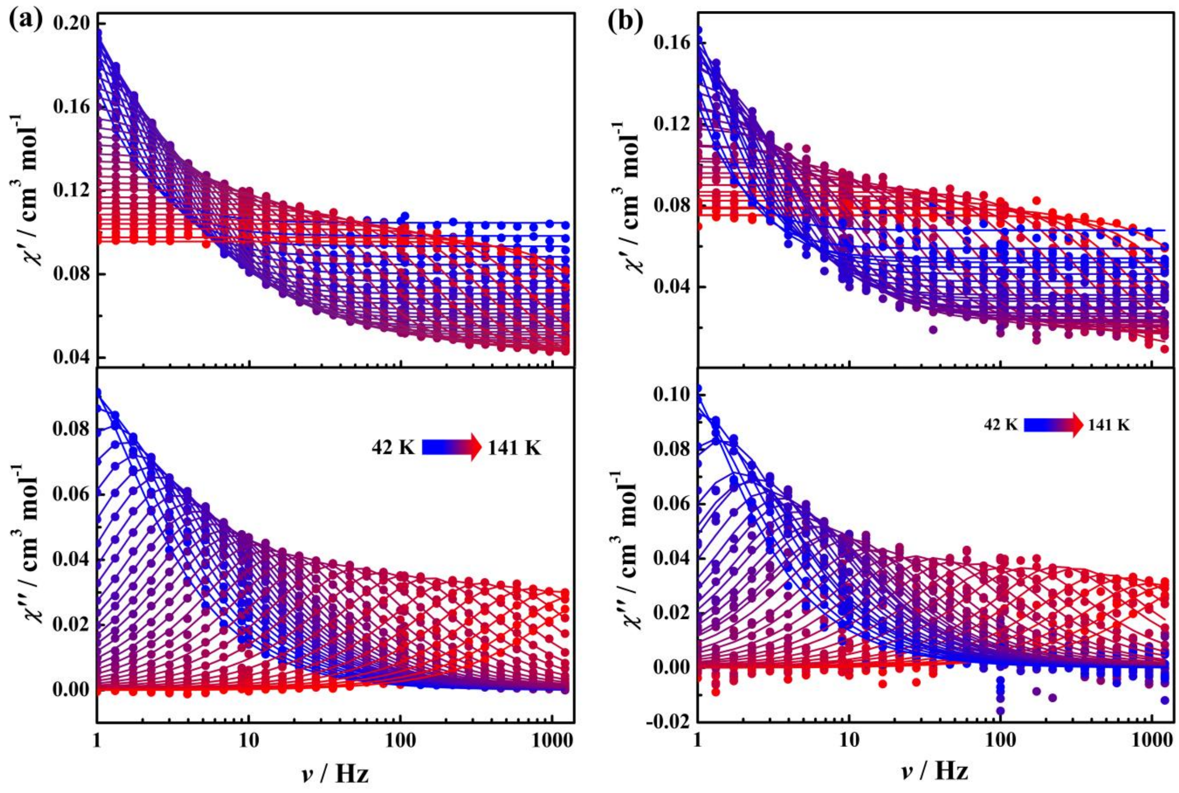

Figure S15. Frequency-dependence of the in-phase (χ', top) and out-of-phase (χ'', bottom) AC susceptibility for **2** (a) and **2@Y** (b) under zero DC field and an oscillating field of 3.5 Oe with ac frequencies of 1–1218 Hz from 42 to 141 K. The solid lines are best fits with Debye model.

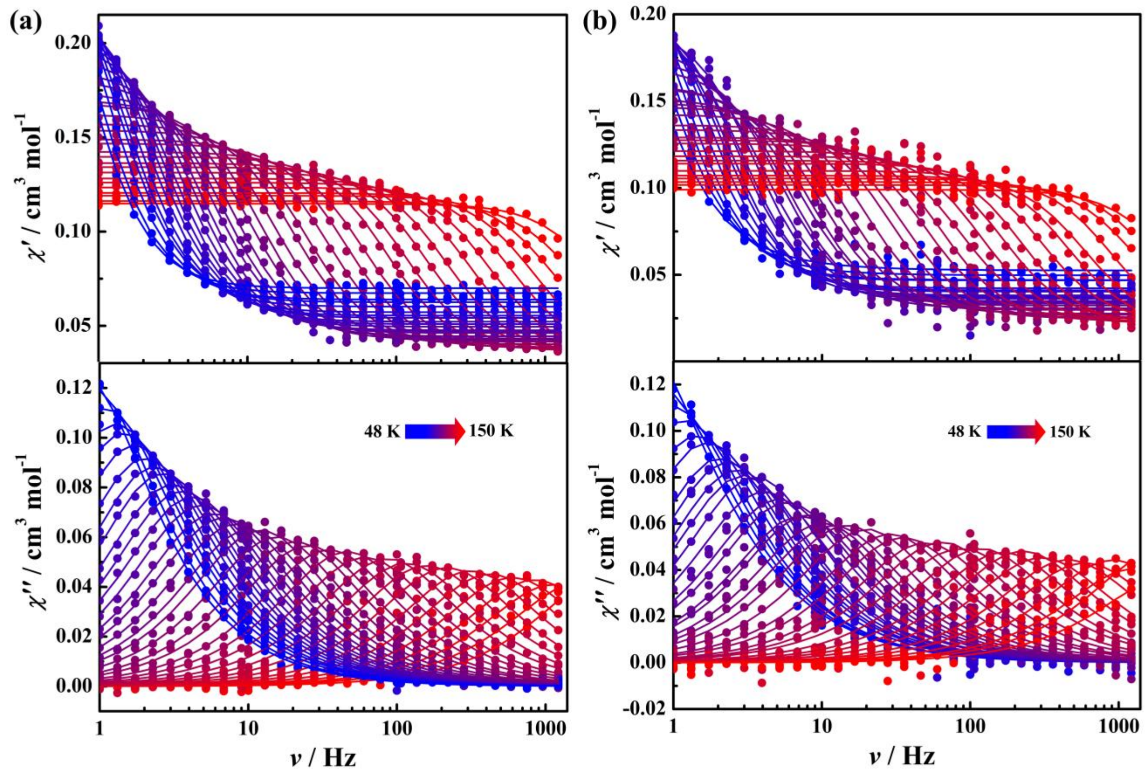

Figure S16. Frequency-dependence of the in-phase (χ', top) and out-of-phase (χ'', bottom) AC susceptibility for **3** (a) and **3@Y** (b) under zero DC field and an oscillating field of 3.5 Oe with ac frequencies of 1–1218 Hz from 48 to 150 K. The solid lines are best fits with Debye model.

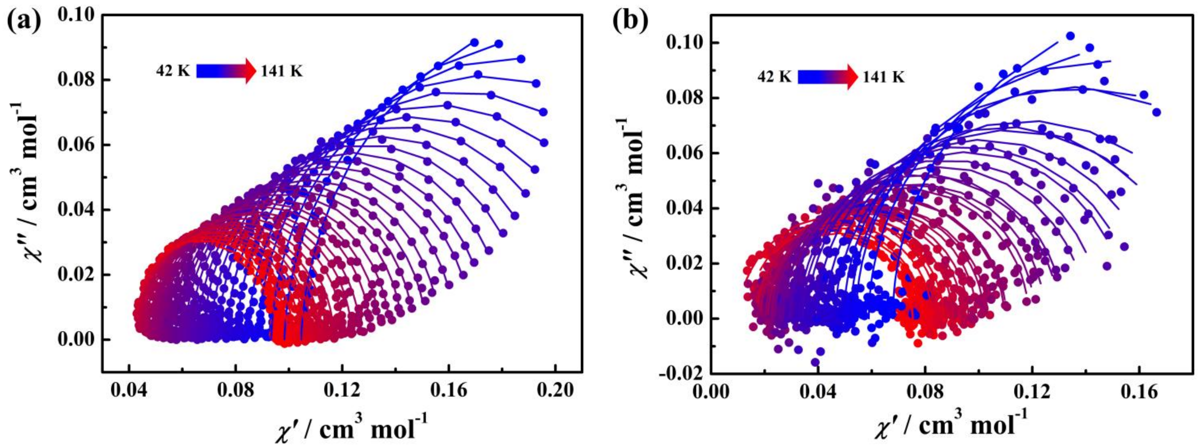

Figure S17. Cole-Cole plots for the ac susceptibilities in zero DC field for **2** (a) and **2@Y** (b) from 42 to 141 K. The solid lines are best fits with Debye model.

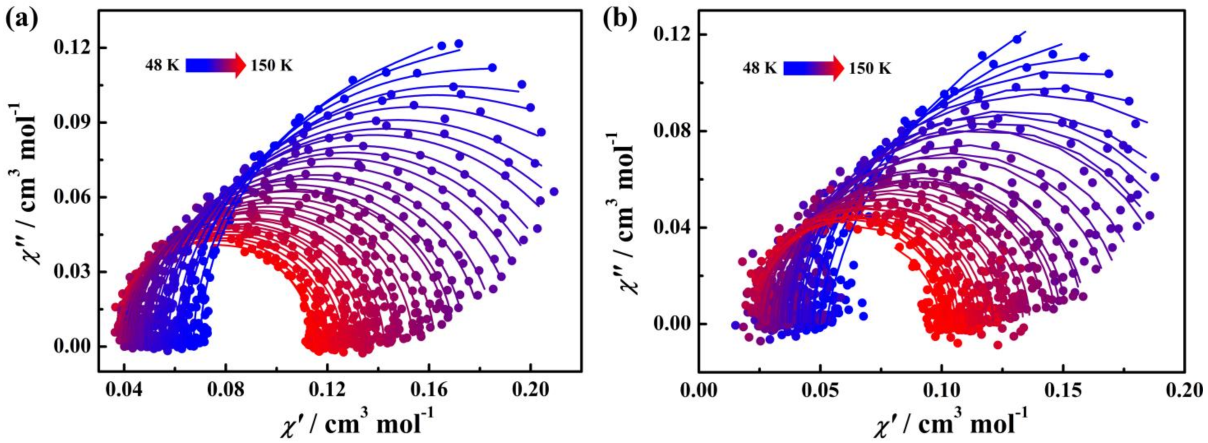

Figure S18. Cole-Cole plots for the ac susceptibilities in zero DC field for **3** (a) and **3@Y** (b) from 48 to 150 K. The solid lines are best fits with Debye model.

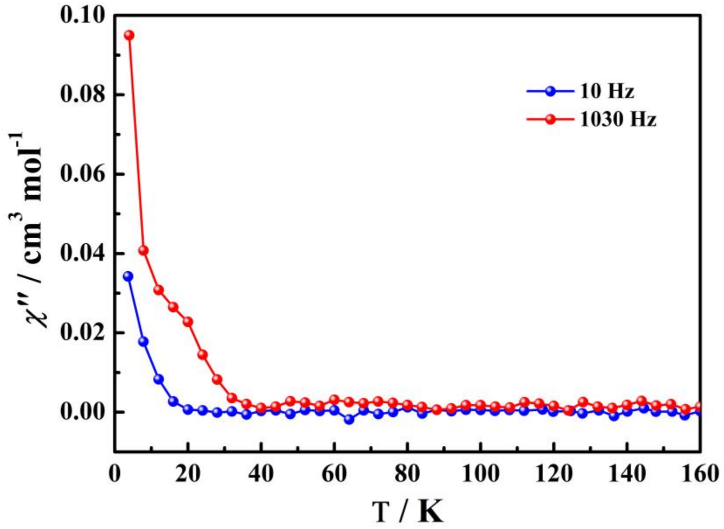

Figure S19. Temperature dependence of the out-of-phase (χ'') ac susceptibility for **2** after one day of exposure to air in a zero DC field with selected AC frequencies of 10 Hz and 1030 Hz.

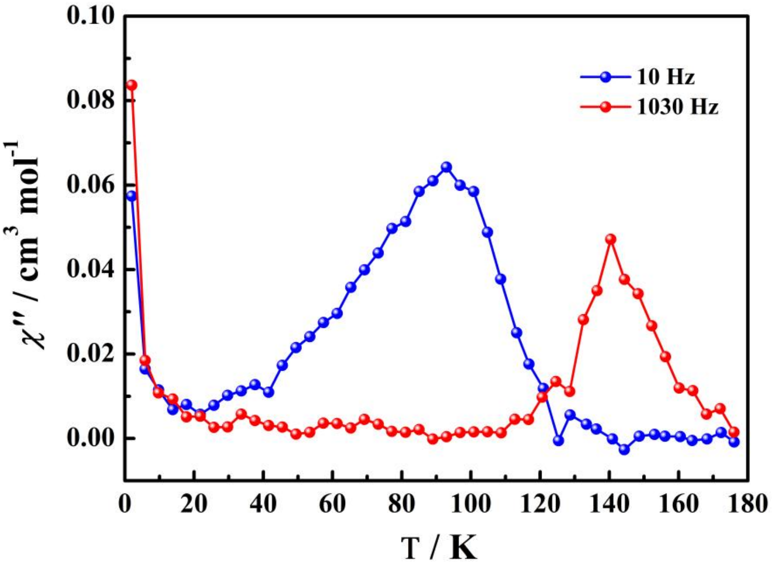

Figure S20. Temperature dependence of the out-of-phase (χ'') ac susceptibility for **3** after one week of exposure to air in a zero DC field with selected AC frequencies of 10 Hz and 1030 Hz.

Table S13. Relaxation fitting parameters of a generalized Debye model for **2**.

T (K)	χ_S (cm^3 mol^{-1})	χ_T (cm^3 mol^{-1})	τ (s)	a	τ_- (s)	τ_+ (s)
42	0.10455	0.31515	0.24906	0.03414	0.15312	0.40512
45	0.09831	0.2909	0.19202	0.02684	0.12505	0.29487
48	0.09335	0.27302	0.1534	0.02296	0.10329	0.22782
51	0.08831	0.25766	0.1252	0.02587	0.0822	0.1907
54	0.08445	0.24319	0.10345	0.02365	0.06923	0.15458
57	0.08026	0.23004	0.08621	0.02219	0.05845	0.12715
60	0.07668	0.21712	0.07255	0.0139	0.05345	0.09848
63	0.07349	0.20689	0.06189	0.00873	0.04862	0.07878
66	0.07065	0.19796	0.0531	0.01024	0.04088	0.06898
69	0.06795	0.18965	0.04631	0.00775	0.0369	0.05812
72	0.06582	0.18141	0.03982	0.00231	0.03519	0.04505
75	0.0632	0.17456	0.03481	0.00485	0.0291	0.04165
78	0.06109	0.16897	0.03067	0.00587	0.02518	0.03736
81	0.05932	0.16251	0.02679	0.00306	0.02324	0.03088
84	0.05746	0.15671	0.02329	0.00486	0.01946	0.02787
87	0.05533	0.15171	0.01988	0.00646	0.01616	0.02446
90	0.05412	0.14697	0.01692	0.00232	0.01495	0.01915
93	0.05238	0.14257	0.01415	0.00249	0.01245	0.01609
96	0.05099	0.13805	0.01175	0.00371	0.01005	0.01374
99	0.04949	0.13427	0.00941	0.00505	0.00784	0.0113
102	0.04831	0.13072	0.00736	0.00261	0.00645	0.00839
105	0.04717	0.12686	0.00565	0.00666	0.00458	0.00697
108	0.04574	0.12358	0.00418	0.00376	0.00357	0.00489
111	0.04469	0.12046	0.00302	0.00583	0.00248	0.00368
114	0.04351	0.11686	0.00211	0.00755	0.00169	0.00264
117	0.04253	0.11394	0.00148	0.00698	0.00119	0.00184
120	0.04197	0.11149	0.00103	0.00445	8.6751E-4	0.00122
123	0.04148	0.10861	6.96154E-4	9.83119E-8	6.95594E-4	6.96714E-4
126	0.0403	0.10616	4.7036E-4	1.83691E-7	4.69843E-4	4.70877E-4
129	0.04002	0.10409	3.2873E-4	2.31743E-7	3.28324E-4	3.29136E-4
132	0.0391	0.10164	2.20766E-4	3.94718E-7	2.20411E-4	2.21122E-4
135	0.03873	0.09966	1.52078E-4	6.21193E-7	1.51771E-4	1.52386E-4
138	0.03927	0.09761	1.07664E-4	9.2978E-7	1.07398E-4	1.07931E-4
141	0.04038	0.09567	7.92745E-5	1.00105E-6	7.90713E-5	7.94782E-5

Table S14. Relaxation fitting parameters of a generalized Debye model for **2@Y**.

T (K)	χ_S ($cm^3\ mol^{-1}$)	χ_T ($cm^3\ mol^{-1}$)	τ (s)	a	τ_- (s)	τ_+ (s)
42	0.06791	0.29248	0.25891	1.05258E-10	0.2589	0.25892
45	0.05893	0.25412	0.19382	7.02563E-11	0.19382	0.19382
48	0.05417	0.24088	0.15629	5.82779E-11	0.15629	0.15629
51	0.05216	0.22008	0.12097	6.20605E-11	0.12097	0.12097
54	0.0497	0.21701	0.10796	6.12016E-11	0.10796	0.10796
57	0.04641	0.18992	0.08629	9.57253E-11	0.08629	0.08629
60	0.04281	0.17958	0.07312	1.05149E-10	0.07312	0.07312
63	0.03965	0.17879	0.06491	8.286E-11	0.06491	0.06491
66	0.03613	0.16589	0.05444	8.85251E-11	0.05444	0.05444
69	0.03302	0.15806	0.04518	7.19581E-11	0.04518	0.04518
72	0.03427	0.15509	0.04062	5.74349E-11	0.04062	0.04062
75	0.03006	0.1453	0.03487	6.91649E-11	0.03487	0.03487
78	0.02928	0.13978	0.03195	6.62479E-11	0.03195	0.03195
81	0.02698	0.13159	0.02812	7.0161E-11	0.02812	0.02812
84	0.02566	0.12984	0.02451	7.38772E-11	0.02451	0.02451
87	0.02688	0.1248	0.02106	6.69655E-11	0.02106	0.02106
90	0.02339	0.12097	0.01919	5.34676E-11	0.01919	0.01919
93	0.02392	0.11864	0.0157	5.24366E-11	0.0157	0.0157
96	0.02302	0.11026	0.01263	6.29646E-11	0.01263	0.01263
99	0.02253	0.1095	0.01013	5.22586E-11	0.01013	0.01013
102	0.02041	0.1019	0.00778	7.23609E-11	0.00778	0.00778
105	0.02128	0.10328	0.00588	5.52039E-11	0.00588	0.00588
108	0.01854	0.09912	0.00426	4.3802E-11	0.00426	0.00426
111	0.01832	0.09609	0.00333	3.50254E-11	0.00333	0.00333
114	0.0173	0.09388	0.00226	3.39597E-11	0.00226	0.00226
117	0.01663	0.09015	0.00161	4.41093E-11	0.00161	0.00161
120	0.01544	0.09011	0.0011	4.56867E-11	0.0011	0.0011
123	0.01041	0.08489	6.64251E-4	5.12569E-11	6.64239E-4	6.64263E-4
126	0.01381	0.08683	5.05856E-4	4.20948E-11	5.05848E-4	5.05864E-4
129	0.01393	0.08248	3.2733E-4	3.59822E-11	3.27325E-4	3.27335E-4
132	0.01303	0.07918	2.32743E-4	2.99618E-11	2.3274E-4	2.32746E-4
135	0.0166	0.07859	1.64358E-4	2.36347E-11	1.64356E-4	1.6436E-4
138	0.01416	0.07541	1.14211E-4	1.7669E-11	1.1421E-4	1.14212E-4
141	0.00734	0.07526	6.65963E-5	0.03486	4.0722E-5	1.08911E-4

Table S15. Relaxation fitting parameters of a generalized Debye model for **3**.

T **(K)**	χ_S **(cm³ mol⁻¹)**	χ_T **(cm³ mol⁻¹)**	τ **(s)**	***a***	τ_- **(s)**	τ_+ **(s)**
48	0.06995	0.3197	0.20945	1.43977E-07	0.20925	0.20965
51	0.06735	0.30755	0.18105	1.4798E-07	0.18087	0.18123
54	0.06396	0.28851	0.1454	1.65167E-07	0.14525	0.14555
57	0.06254	0.27434	0.12282	1.48178E-07	0.1227	0.12294
60	0.06017	0.26476	0.10886	1.51595E-07	0.10875	0.10897
63	0.05722	0.25213	0.09253	1.70104E-07	0.09243	0.09263
66	0.05542	0.23978	0.0778	1.85045E-07	0.07771	0.07789
69	0.05419	0.23142	0.06781	1.89119E-07	0.06773	0.06789
72	0.05217	0.22426	0.05942	2.3476E-07	0.05935	0.05949
75	0.05089	0.2141	0.0513	2.58539E-07	0.05123	0.05137
78	0.0495	0.20736	0.04423	2.75146E-07	0.04417	0.04429
81	0.04763	0.20074	0.03774	2.87926E-07	0.03769	0.03779
84	0.04701	0.19358	0.03274	3.13591E-07	0.03269	0.03279
87	0.04618	0.1858	0.02714	3.3464E-07	0.0271	0.02718
90	0.04442	0.18078	0.02338	3.3856E-07	0.02335	0.02341
93	0.04267	0.17395	0.01926	3.60031E-07	0.01923	0.01929
96	0.04324	0.16999	0.0163	3.87077E-07	0.01627	0.01633
99	0.04169	0.16771	0.0137	3.08226E-07	0.01368	0.01372
102	0.04158	0.16215	0.01124	3.45434E-07	0.01122	0.01126
105	0.04022	0.15716	0.00887	2.28076E-07	0.00886	0.00888
108	0.04013	0.15374	0.00688	2.52954E-07	0.00687	0.00689
111	0.03858	0.14898	0.00547	2.27643E-07	0.00546	0.00548
114	0.038	0.14673	0.00403	2.03331E-07	0.00403	0.00403
117	0.03818	0.14228	0.003	2.32078E-07	0.003	0.003
120	0.03679	0.1397	0.00217	1.90453E-07	0.00217	0.00217
123	0.03707	0.13633	0.00154	2.21653E-07	0.00154	0.00154
126	0.03676	0.13422	0.00104	1.89339E-07	0.00104	0.00104
129	0.03554	0.13133	7.32778E-4	1.48199E-07	7.32055E-4	7.33502E-4
132	0.03495	0.12818	5.09758E-4	1.438E-07	5.09262E-4	5.10254E-4
135	0.03525	0.12597	3.74813E-4	1.27251E-07	3.7447E-4	3.75156E-4
138	0.03551	0.12337	2.66153E-4	1.10759E-07	2.65926E-4	2.6638E-4
141	0.03458	0.1205	1.88634E-4	9.18842E-08	1.88487E-4	1.88781E-4
144	0.03693	0.11904	1.41331E-4	8.65187E-08	1.41224E-4	1.41438E-4
147	0.02805	0.11624	8.98781E-5	0.02709	5.84082E-5	1.38304E-4
150	0.0243	0.11479	6.10403E-5	0.0088	4.79091E-5	7.77706E-5

Table S16. Relaxation fitting parameters of a generalized Debye model for **3@Y**.

T **(K)**	χ_S **(cm^3 mol^{-1})**	χ_T **(cm^3 mol^{-1})**	***τ*** **(s)**	***a***	τ_- **(s)**	τ_+ **(s)**
48	0.05249	0.38327	0.32038	0.08966	0.14046	0.73077
51	0.04968	0.30886	0.20799	0.05147	0.11351	0.3811
54	0.04697	0.28059	0.16389	0.03195	0.10245	0.26218
57	0.04679	0.25816	0.13395	0.00446	0.1128	0.15907
60	0.04223	0.24469	0.10968	0.01901	0.07661	0.15702
63	0.04101	0.23577	0.0932	0.01411	0.0685	0.12681
66	0.04034	0.21728	0.07646	4.63E-8	0.07642	0.0765
69	0.03727	0.21087	0.0681	7.34449E-8	0.06805	0.06815
72	0.03812	0.20521	0.05909	1.05298E-7	0.05904	0.05914
75	0.03629	0.19801	0.0536	1.58774E-7	0.05355	0.05365
78	0.0341	0.19488	0.04465	2.4356E-7	0.04459	0.04471
81	0.03488	0.18329	0.03887	3.75911E-7	0.03881	0.03893
84	0.0335	0.17515	0.03102	5.92221E-7	0.03096	0.03108
87	0.03071	0.16964	0.02736	9.01934E-7	0.02729	0.02743
90	0.03234	0.15939	0.02209	1.26949E-6	0.02203	0.02215
93	0.02979	0.15799	0.01956	1.82814E-6	0.01949	0.01963
96	0.0304	0.15133	0.01575	3.23252E-6	0.01568	0.01582
99	0.0301	0.14896	0.01335	4.99284E-6	0.01327	0.01343
102	0.02767	0.14665	0.01078	8.10563E-6	0.0107	0.01086
105	0.02693	0.14221	0.00865	1.32109E-5	0.00857	0.00873
108	0.02486	0.13617	0.0071	1.98967E-5	0.00702	0.00718
111	0.02507	0.13345	0.0057	2.99113E-5	0.00562	0.00578
114	0.02547	0.12911	0.00432	4.71059E-5	0.00424	0.0044
117	0.02325	0.1275	0.00311	8.17902E-5	0.00304	0.00318
120	0.02188	0.12567	0.00226	0.03213	0.00141	0.00362
123	0.0226	0.12121	0.00154	2.33782E-16	0.00154	0.00154
126	0.02271	0.11826	0.00101	3.03815E-16	0.00101	0.00101
129	0.01926	0.11576	7.05302E-4	4.18117E-16	7.05302E-4	7.05302E-4
132	0.02307	0.11397	5.27223E-4	5.2554E-16	5.27223E-4	5.27223E-4
135	0.02068	0.10984	3.49031E-4	7.14475E-16	3.49031E-4	3.49031E-4
138	0.0207	0.10708	2.69159E-4	9.87892E-16	2.69159E-4	2.69159E-4
141	0.01663	0.10544	1.73795E-4	1.76824E-15	1.73795E-4	1.73795E-4
144	0.0223	0.10345	1.27112E-4	2.48547E-15	1.27112E-4	1.27112E-4
147	0.00457	0.10172	8.31355E-5	2.79535E-15	8.31355E-5	8.31355E-5
150	0.02611	0.09898	7.54188E-5	3.63676E-18	7.54188E-5	7.54188E-5

Table S17. All Dy-based SMMs with U_{eff} above 2000 K.

Compound	air stability	U_{eff}(K)	Ref.
$[Dy(18\text{-}C\text{-}6)(1\text{-}AdO)_2][I_3]$ **(3)**	stable	2427	This work
$[Dy(18\text{-}C\text{-}6)(OtBu)_2][I_3]$ **(2)**	unstable	2352	This work
$(Cp^{iPr5})_2Dy_2I_3$	unstable	2347	1
$[K(2.2.2)][[1\text{-(piperidino)-}2,3,4,5\text{-tetraphenylborolyl}]_2Dy]$	unstable	2300	2
$[(Cp^{iPr5})Dy(Cp^*)][B(C_6F_5)_4]$	unstable	2215	3
$[K(18\text{-crown-}6)][Dy(BC_4Ph_5)_2]$	unstable	2156	4
$[(Cp^{ipr4Me)})_2Dy][B(C_6F_5)_4]$	unstable	2110	5

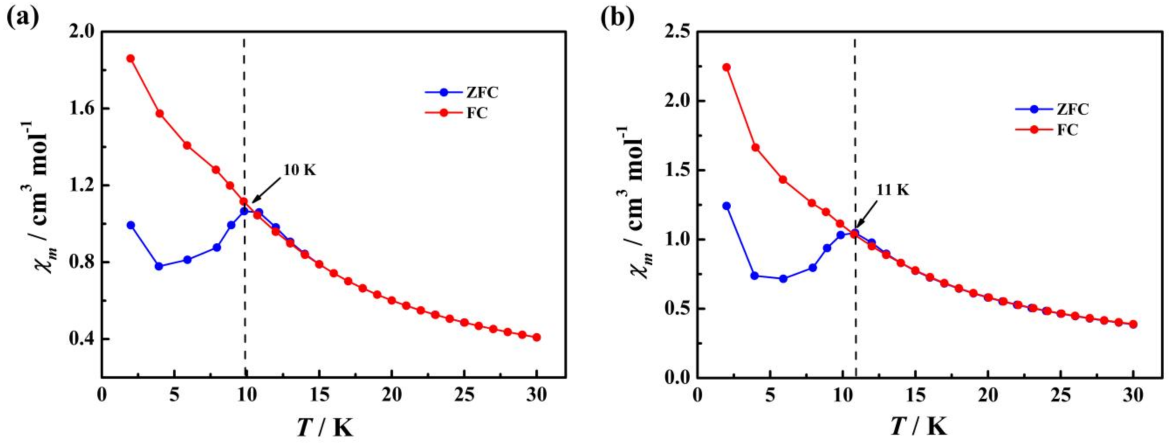

Figure S21. Field-cooled (FC, red) in cool mode from 30 to 2 K and zero-field-cooled (ZFC, blue) in warm mode from 2 to 30 K magnetic susceptibility for **2** (a) and **2@Y** (b) under an applied DC field of 2000 Oe.

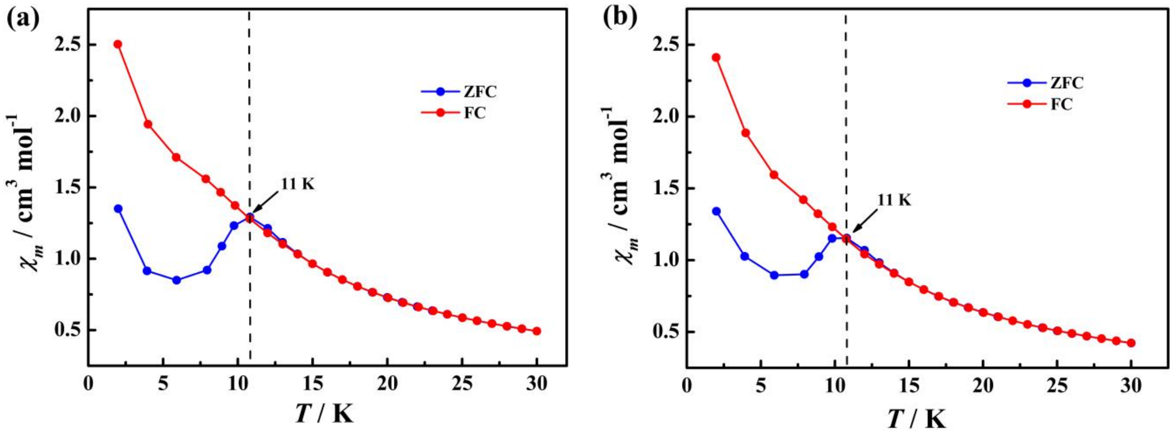

Figure S22. Field-cooled (FC, red) in cool mode from 30 to 2 K and zero-field-cooled (ZFC, blue) in warm mode from 2 to 30 K magnetic susceptibility for **3** (a) and **3@Y** (b) under an applied DC field of 2000 Oe.

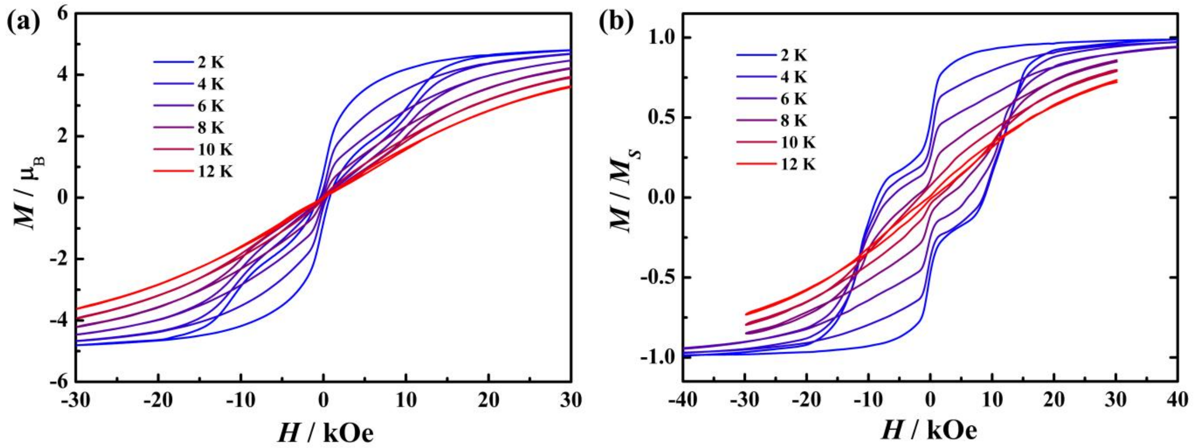

Figure S23. Magnetic hysteresis loops for **2** (a) and **2@Y** (b). The data were collected at temperature range of 2 to 12 K with an average sweep rate of 15 Oe s^{-1}.

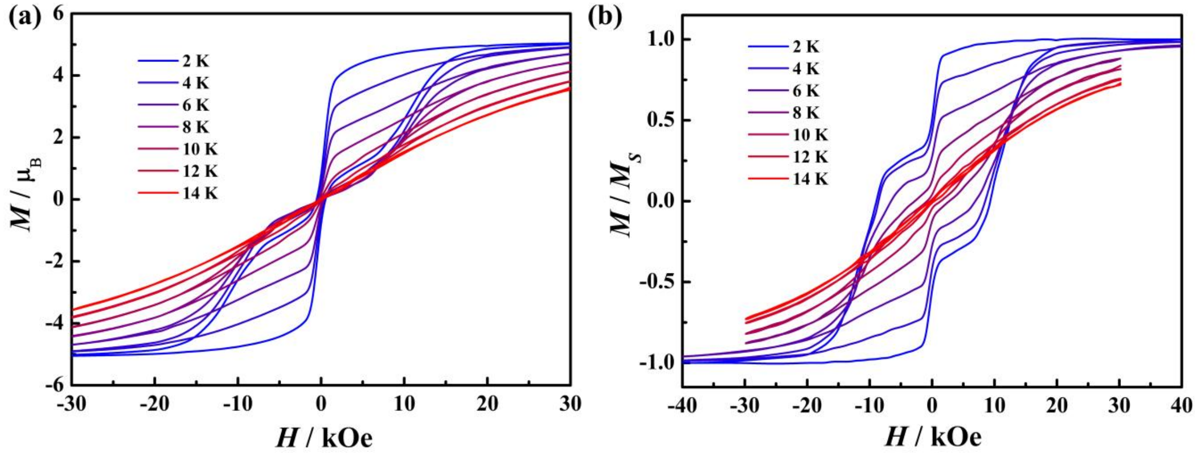

Figure S24. Magnetic hysteresis loops for **3** (a) and **3@Y** (b). The data were collected at temperature range of 2 to 14 K with an average sweep rate of 15 Oe s^{-1}.

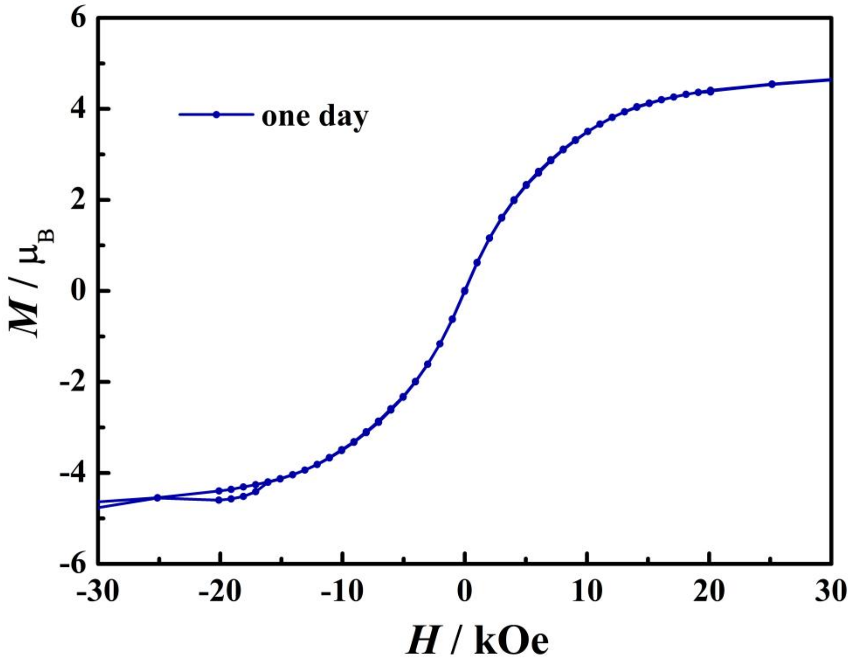

Figure S25. Magnetic hysteresis loops of **2** measured after one day of exposure to air. The data were collected at 2 K with an average sweep rate of 15 Oe s^{-1}.

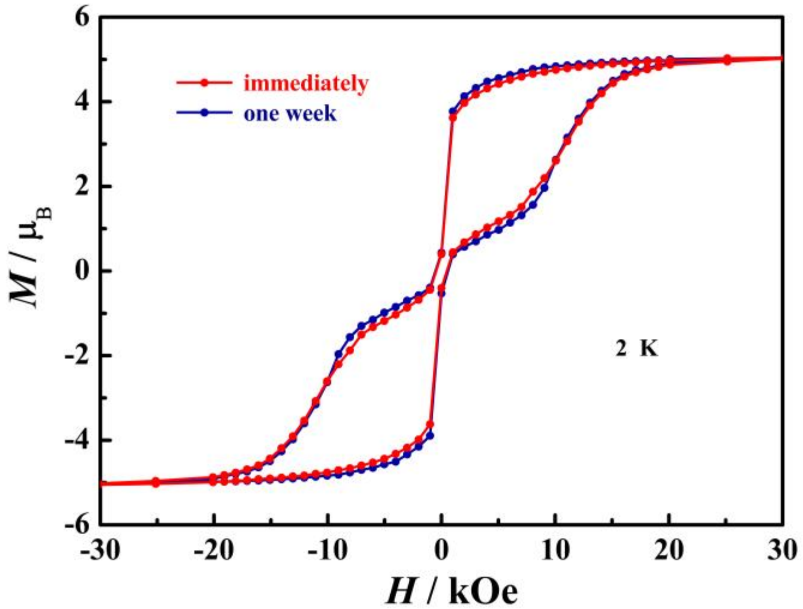

Figure S26. Magnetic hysteresis loops of **3** measured immediately (red) and after one week of exposure to air (dark blue). The data were collected at 2 K with an average sweep rate of 15 Oe s^{-1}.

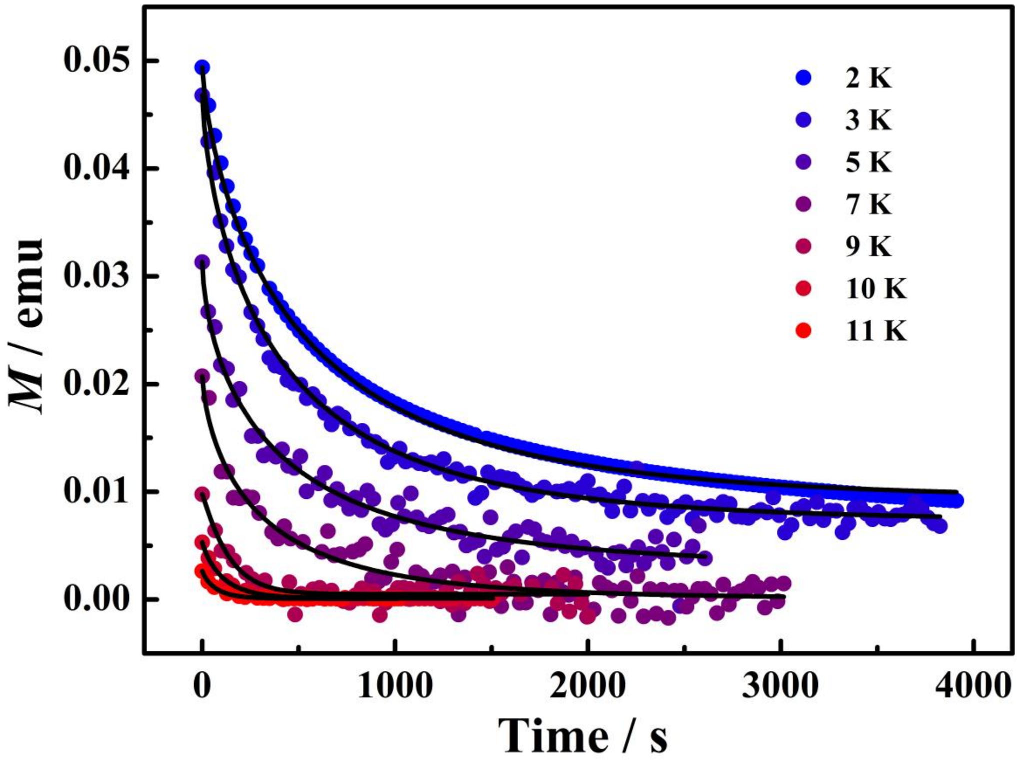

Figure S27. Plot of magnetization decay vs. time used to extract relaxation times τ for **2@Y** at 2, 3, 5, 7, 9, 10 and 11 K. Red line represents the fits to the data using the equation $M(\mathrm{t}) = M_{eq} + (M_0 - M_{eq})\exp[-(\mathrm{t}/\tau)^{\beta}]$, where M_0 is the initial magnetization after zero external field was achieved.

Table S18. Parameters used to fit dc magnetic relaxation data for **2@Y** and magnetic relaxation times extracted from these fits.

T **(K)**	$\boldsymbol{M_0}$ **(emu)**	$\boldsymbol{M_{eq}}$ **(emu)**	$\boldsymbol{\beta}$	$\boldsymbol{\tau}$ **(s)**
2	0.049395	0.009186(4.13×10^{-4})	0.7242(0.0273)	562.4(20.4)
3	0.046789	0.007295(27.56×10^{-4})	0.6949(0.0615)	425.2(14.3)
5	0.031320	0.00298(2.146×10^{-4})	0.6457(0.159)	405.8(61.9)
7	0.020738	9.13×10^{-4}(7.474×10^{-4})	0.6883(0.2257)	311.3(40.5)
9	0.009762	4.806×10^{-4}(2.465×10^{-4})	0.9946(0.5470)	137.7(62.1)
10	0.005314	1.041×10^{-4}(2.465×10^{-5})	0.9062(0.0430)	107.6(3.9)
11	0.002642	7.91×10^{-5}(7.41×10^{-6})	0.9012(0.0239)	72.6(1.43)

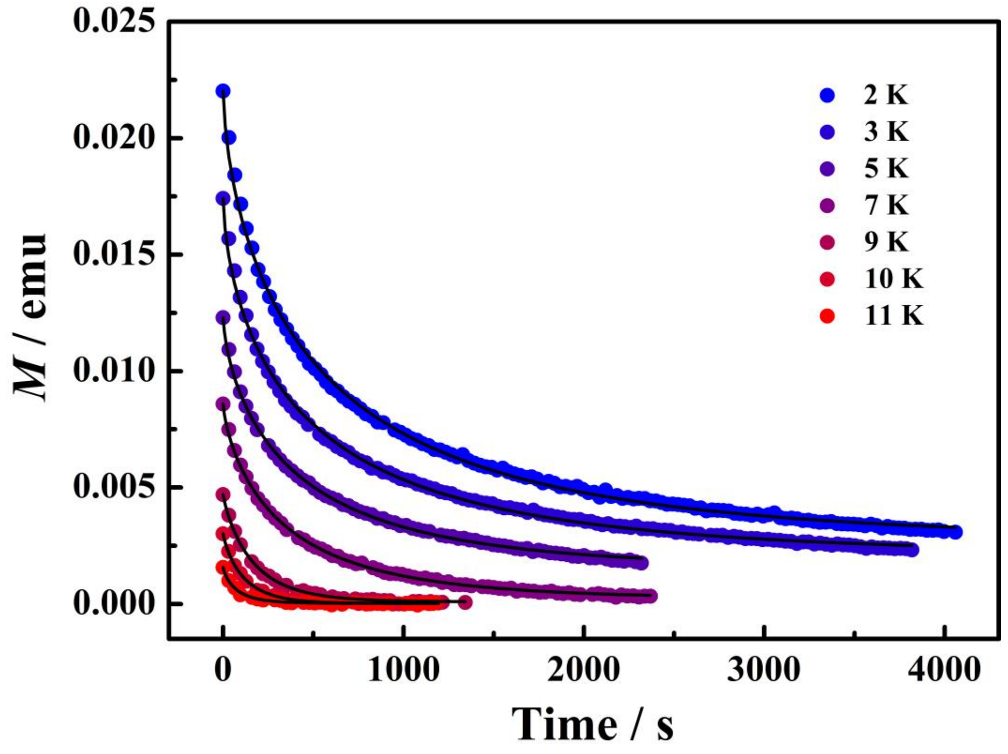

Figure S28. Plot of magnetization decay vs. time used to extract relaxation times τ for **3@Y** at 2, 3, 5, 7, 9, 10 and 11 K. Red line represents the fits to the data using the equation $M(t) = M_{eq} + (M_0 - M_{eq})\exp[-(t/\tau)^{\beta}]$, where M_0 is the initial magnetization after zero external field was achieved.

Table S19. Parameters used to fit dc magnetic relaxation data for **3@Y** and magnetic relaxation times extracted from these fits.

T **(K)**	$\boldsymbol{M_0}$ **(emu)**	$\boldsymbol{M_{eq}}$ **(emu)**	$\boldsymbol{\beta}$	$\boldsymbol{\tau}$ **(s)**
2	0.022023	0.002563(2.79×10^{-4})	0.631(0.0257)	587.8(30.0)
3	0.017412	0.002098(2.03×10^{-4})	0.6317(0.0263)	497.8(24.5)
5	0.012298	0.001526(2.19×10^{-4})	0.6846(0.0361)	422.8(27.7)
7	0.008590	2.176×10^{-4}(8.84×10^{-5})	0.7316(0.272)	353.3(13.1)
9	0.004692	9.226×10^{-5}(5.432×10^{-5})	0.8784(0.054)	166.9(8.0)
10	0.003010	1.5×10^{-5}(3.5517×10^{-5})	0.8693(0.0774)	116.3(8.1)
11	0.001570	2.5×10^{-5}(3.8743×10^{-5})	0.8463(0.1567)	94.4(14.1)

4. *Ab initio* Calculation

Table S20. SA-CASSCF/RASSI calculated electronic states for **2**.

Ab initio **Energy (cm^{-1})**	***Ab initio*** **Energy (K)**	g_x	g_y	g_z	**g_z Angle (°)**	**Crystal field Wavefunction**
0	0	0.00	0.00	19.89	-	100%|±15/2>
657	945	0.03	0.03	16.91	0.17	99.9%|±13/2>
1174	1688	0.00	0.02	14.06	0.90	99.4%|±11/2>
1511	2172	0.21	0.23	10.78	83.72	54.1%|±9/2>+40.8%|∓9/2>
1659	2385	9.09	8.95	4.32	3.19	25.9%|±5/2>+62.2%|∓7/2>
1727	2483	1.11	6.76	13.92	0.46	83.3%|±1/2>+12.1%|∓1/2>
1753	2520	2.04	2.98	5.18	2.98	90.4%|∓3/2>
1787	2569	10.31	7.76	1.74	2.48	68.0%|±5/2>+29.1%|∓7/2>

Only components with > 10% contribution are given, rounded to the nearest percent.

Table S21. SA-CASSCF/RASSI calculated electronic states for **3**.

Ab initio **Energy (cm^{-1})**	***Ab initio*** **Energy (K)**	g_x	g_y	g_z	**g_z Angle (°)**	**Crystal field Wavefunction**
0	0	0.00	0.00	19.90	-	100%|±15/2>
673	968	0.02	0.02	16.90	0.77	99.9%|±13/2>
1211	1741	0.01	0.03	14.06	1.12	99.5%|±11/2>
1567	2253	0.14	0.14	10.88	0.32	87.8%|±9/2>
1726	2481	9.09	8.75	4.50	5.00	69.6%|±7/2>+27.0%|∓5/2>
1785	2566	11.51	9.56	1.27	2.37	92.1%|±1/2>
1812	2605	3.21	2.80	0.91	76.27	91.4%|∓3/2>
1848	2657	10.89	7.01	1.82	5.99	69.0%|±5/2>+26.5%|∓7/2>

Only components with > 10% contribution are given, rounded to the nearest percent.

Table S22. *Ab initio* calculated crystal field parameters for Dy(III) ion in **2**.

Crystal Field Parameter B_k^q		Value / cm^{-1}
k	*q*	
2	-2	-2.6909199972663E-02
2	-1	-3.3796617448393E-03
2	0	-1.0256887695238E+01
2	1	4.0385586821537E-02
2	2	1.1818390589346E-01
4	-4	-1.2617350921532E-04
4	-3	-6.9243756655159E-04
4	-2	5.4842607216817E-04
4	-1	5.7025094727512E-04
4	0	-1.4695595785908E-02
4	1	-1.4424643207962E-03
4	2	-1.1180176103074E-04
4	3	-6.1307629440651E-03
4	4	-5.6030470728628E-04
6	-6	5.2995542961907E-04
6	-5	-4.9811717869502E-05
6	-4	-6.9095778071612E-07
6	-3	-2.2402885078896E-06
6	-2	-7.1370983672897E-07
6	-1	9.0475464297917E-06
6	0	3.8488244803232E-05
6	1	1.0990991560598E-05
6	2	4.4651477107221E-07
6	3	-4.9501390734242E-06
6	4	-4.4059044057635E-06
6	5	1.3002662942113E-05
6	6	4.6902241519379E-05

Table S23. Average transition magnetic moment elements between the states in **2**, given in μ_B

<table>
<tr><th>Matrix Element
< Mult. i | Mult. j ></th><th>Average Value</th></tr>
<tr><td>< 1.1+ | 1.1- ></td><td>7.81E-06</td></tr>
<tr><td>< 2.1+ | 2.1- ></td><td>1.01E-02</td></tr>
<tr><td>< 3.1+ | 3.1- ></td><td>4.99E-03</td></tr>
<tr><td>< 4.1+ | 4.1- ></td><td>7.42E-02</td></tr>
<tr><td>< 5.1+ | 5.1- ></td><td>3.00E+00</td></tr>
<tr><td>< 6.1+ | 6.1- ></td><td>3.50E+00</td></tr>
<tr><td>< 7.1+ | 7.1- ></td><td>1.25E+00</td></tr>
<tr><td>< 8.1+ | 8.1- ></td><td>2.88E+00</td></tr>
<tr><td>< 1.1+ | 2.1+ ></td><td>1.79E+00</td></tr>
<tr><td>< 1.1+ | 2.1- ></td><td>3.64E-05</td></tr>
<tr><td>< 2.1+ | 3.1+ ></td><td>2.40E+00</td></tr>
<tr><td>< 2.1+ | 3.1- ></td><td>1.34E-02</td></tr>
<tr><td>< 3.1+ | 4.1+ ></td><td>2.77E+00</td></tr>
<tr><td>< 3.1+ | 4.1- ></td><td>5.00E-03</td></tr>
<tr><td>< 4.1+ | 5.1+ ></td><td>2.92E+00</td></tr>
<tr><td>< 4.1+ | 5.1- ></td><td>1.25E-01</td></tr>
<tr><td>< 5.1+ | 6.1+ ></td><td>3.32E-01</td></tr>
<tr><td>< 5.1+ | 6.1- ></td><td>3.13E-01</td></tr>
<tr><td>< 6.1+ | 7.1+ ></td><td>3.25E+00</td></tr>
<tr><td>< 6.1+ | 7.1- ></td><td>3.66E-01</td></tr>
<tr><td>< 7.1+ | 8.1+ ></td><td>3.10E+00</td></tr>
<tr><td>< 7.1+ | 8.1- ></td><td>6.12E-01</td></tr>
<tr><td>< 1.1+ | 3.1+ ></td><td>1.06E-02</td></tr>
<tr><td>< 1.1+ | 3.1- ></td><td>3.71E-03</td></tr>
<tr><td>< 2.1+ | 4.1+ ></td><td>1.66E-02</td></tr>
<tr><td>< 2.1+ | 4.1- ></td><td>2.20E-02</td></tr>
<tr><td>< 3.1+ | 5.1+ ></td><td>3.05E-02</td></tr>
<tr><td>< 3.1+ | 5.1- ></td><td>4.46E-02</td></tr>
<tr><td>< 4.1+ | 6.1+ ></td><td>1.78E-01</td></tr>
<tr><td>< 4.1+ | 6.1- ></td><td>6.06E-01</td></tr>
<tr><td>< 5.1+ | 7.1+ ></td><td>2.20E-01</td></tr>
<tr><td>< 5.1+ | 7.1- ></td><td>1.30E+00</td></tr>
<tr><td>< 6.1+ | 8.1+ ></td><td>9.04E-02</td></tr>
<tr><td>< 6.1+ | 8.1- ></td><td>4.07E-01</td></tr>
<tr><td>< 1.1+ | 4.1+ ></td><td>3.87E-03</td></tr>
<tr><td>< 1.1+ | 4.1- ></td><td>5.40E-03</td></tr>
<tr><td>< 2.1+ | 5.1+ ></td><td>1.18E-02</td></tr>
<tr><td>< 2.1+ | 5.1- ></td><td>5.91E-03</td></tr>
<tr><td>< 3.1+ | 6.1+ ></td><td>2.07E-01</td></tr>
<tr><td>< 3.1+ | 6.1- ></td><td>2.24E-01</td></tr>
<tr><td>< 4.1+ | 7.1+ ></td><td>1.13E-01</td></tr>
<tr><td>< 4.1+ | 7.1- ></td><td>6.94E-01</td></tr>
<tr><td>< 5.1+ | 8.1+ ></td><td>1.34E+00</td></tr>
<tr><td>< 5.1+ | 8.1- ></td><td>1.30E+00</td></tr>
<tr><td>< 1.1+ | 5.1+ ></td><td>3.19E-03</td></tr>
<tr><td>< 1.1+ | 5.1- ></td><td>1.52E-02</td></tr>
<tr><td>< 2.1+ | 6.1+ ></td><td>7.27E-02</td></tr>
<tr><td>< 2.1+ | 6.1- ></td><td>8.49E-02</td></tr>
<tr><td>< 3.1+ | 7.1+ ></td><td>5.76E-02</td></tr>
<tr><td>< 3.1+ | 7.1- ></td><td>3.56E-01</td></tr>
<tr><td>< 4.1+ | 8.1+ ></td><td>1.76E-01</td></tr>
<tr><td>< 4.1+ | 8.1- ></td><td>9.92E-01</td></tr>
<tr><td>< 1.1+ | 6.1+ ></td><td>1.86E-02</td></tr>
<tr><td>< 1.1+ | 6.1- ></td><td>4.44E-03</td></tr>
<tr><td>< 2.1+ | 7.1+ ></td><td>7.74E-02</td></tr>
<tr><td>< 2.1+ | 7.1- ></td><td>1.69E-02</td></tr>
<tr><td>< 3.1+ | 8.1+ ></td><td>3.58E-02</td></tr>
<tr><td>< 3.1+ | 8.1- ></td><td>2.62E-02</td></tr>
<tr><td>< 1.1+ | 7.1+ ></td><td>2.07E-02</td></tr>
<tr><td>< 1.1+ | 7.1- ></td><td>3.92E-03</td></tr>
<tr><td>< 2.1+ | 8.1+ ></td><td>8.12E-03</td></tr>
<tr><td>< 2.1+ | 8.1- ></td><td>8.59E-03</td></tr>
<tr><td>< 1.1+ | 8.1+ ></td><td>2.20E-02</td></tr>
<tr><td>< 1.1+ | 8.1- ></td><td>4.06E-03</td></tr>
</table>

Table S24. *Ab initio* calculated crystal field parameters for Dy(III) ion in **3**.

**Crystal Field Parameter** B_k^q *k*	*q*	**Value / cm^{-1}**
2	-2	2.5666549329574E-05
2	-1	-1.5822821424316E-05
2	0	-1.0640811702023E+01
2	1	-7.6844816719566E-03
2	2	5.6634944029120E-02
4	-4	1.5303305282498E-06
4	-3	-2.8738540296469E-06
4	-2	-6.7867655411780E-07
4	-1	1.3553451425890E-06
4	0	-1.5140705893304E-02
4	1	-3.6938860911132E-04
4	2	-3.4758501416549E-05
4	3	2.4095276213133E-03
4	4	1.2949934419004E-03
6	-6	1.0412171182104E-06
6	-5	-1.1832215242199E-09
6	-4	9.7530576282068E-09
6	-3	-2.1199491097285E-08
6	-2	-7.1608650681644E-09
6	-1	1.8444861040209E-08
6	0	4.2641561156411E-05
6	1	5.4955074867907E-05
6	2	-7.7955211809220E-06
6	3	-3.3585945057924E-06
6	4	8.2191667036941E-06
6	5	9.8711281969851E-06
6	6	5.0023215931693E-04

Table S25. Average transition magnetic moment elements between the states in **3**, given in μ_B.

<table>
<tr><th>Matrix Element
< Mult. i | Mult. j ></th><th>Average Value</th></tr>
<tr><td>< 1.1+ | 1.1- ></td><td>1.20E-06</td></tr>
<tr><td>< 2.1+ | 2.1- ></td><td>8.18E-03</td></tr>
<tr><td>< 3.1+ | 3.1- ></td><td>6.87E-03</td></tr>
<tr><td>< 4.1+ | 4.1- ></td><td>4.67E-02</td></tr>
<tr><td>< 5.1+ | 5.1- ></td><td>2.96E+00</td></tr>
<tr><td>< 6.1+ | 6.1- ></td><td>3.44E+00</td></tr>
<tr><td>< 7.1+ | 7.1- ></td><td>6.22E-01</td></tr>
<tr><td>< 8.1+ | 8.1- ></td><td>2.71E+00</td></tr>
<tr><td>< 1.1+ | 2.1+ ></td><td>1.79E+00</td></tr>
<tr><td>< 1.1+ | 2.1- ></td><td>5.34E-05</td></tr>
<tr><td>< 2.1+ | 3.1+ ></td><td>2.40E+00</td></tr>
<tr><td>< 2.1+ | 3.1- ></td><td>1.09E-02</td></tr>
<tr><td>< 3.1+ | 4.1+ ></td><td>2.78E+00</td></tr>
<tr><td>< 3.1+ | 4.1- ></td><td>7.18E-03</td></tr>
<tr><td>< 4.1+ | 5.1+ ></td><td>2.94E+00</td></tr>
<tr><td>< 4.1+ | 5.1- ></td><td>1.90E-01</td></tr>
<tr><td>< 5.1+ | 6.1+ ></td><td>8.30E-02</td></tr>
<tr><td>< 5.1+ | 6.1- ></td><td>2.94E-01</td></tr>
<tr><td>< 6.1+ | 7.1+ ></td><td>3.42E+00</td></tr>
<tr><td>< 6.1+ | 7.1- ></td><td>5.78E-01</td></tr>
<tr><td>< 7.1+ | 8.1+ ></td><td>3.06E+00</td></tr>
<tr><td>< 7.1+ | 8.1- ></td><td>8.01E-01</td></tr>
<tr><td>< 1.1+ | 3.1+ ></td><td>3.05E-02</td></tr>
<tr><td>< 1.1+ | 3.1- ></td><td>3.06E-03</td></tr>
<tr><td>< 2.1+ | 4.1+ ></td><td>2.68E-02</td></tr>
<tr><td>< 2.1+ | 4.1- ></td><td>1.78E-02</td></tr>
<tr><td>< 3.1+ | 5.1+ ></td><td>8.18E-03</td></tr>
<tr><td>< 3.1+ | 5.1- ></td><td>2.19E-02</td></tr>
<tr><td>< 4.1+ | 6.1+ ></td><td>5.73E-02</td></tr>
<tr><td>< 4.1+ | 6.1- ></td><td>6.14E-01</td></tr>
<tr><td>< 5.1+ | 7.1+ ></td><td>3.61E-01</td></tr>
<tr><td>< 5.1+ | 7.1- ></td><td>1.42E+00</td></tr>
<tr><td>< 6.1+ | 8.1+ ></td><td>9.17E-02</td></tr>
<tr><td>< 6.1+ | 8.1- ></td><td>2.40E-01</td></tr>
<tr><td>< 1.1+ | 4.1+ ></td><td>3.69E-03</td></tr>
<tr><td>< 1.1+ | 4.1- ></td><td>4.66E-03</td></tr>
<tr><td>< 2.1+ | 5.1+ ></td><td>5.80E-03</td></tr>
<tr><td>< 2.1+ | 5.1- ></td><td>4.75E-03</td></tr>
<tr><td>< 3.1+ | 6.1+ ></td><td>1.87E-01</td></tr>
<tr><td>< 3.1+ | 6.1- ></td><td>2.37E-01</td></tr>
<tr><td>< 4.1+ | 7.1+ ></td><td>1.26E-01</td></tr>
<tr><td>< 4.1+ | 7.1- ></td><td>7.51E-01</td></tr>
<tr><td>< 5.1+ | 8.1+ ></td><td>1.38E+00</td></tr>
<tr><td>< 5.1+ | 8.1- ></td><td>1.34E+00</td></tr>
<tr><td>< 1.1+ | 5.1+ ></td><td>2.44E-03</td></tr>
<tr><td>< 1.1+ | 5.1- ></td><td>1.32E-02</td></tr>
<tr><td>< 2.1+ | 6.1+ ></td><td>7.14E-02</td></tr>
<tr><td>< 2.1+ | 6.1- ></td><td>7.58E-02</td></tr>
<tr><td>< 3.1+ | 7.1+ ></td><td>4.23E-02</td></tr>
<tr><td>< 3.1+ | 7.1- ></td><td>3.45E-01</td></tr>
<tr><td>< 4.1+ | 8.1+ ></td><td>2.67E-01</td></tr>
<tr><td>< 4.1+ | 8.1- ></td><td>1.01E+00</td></tr>
<tr><td>< 1.1+ | 6.1+ ></td><td>1.84E-02</td></tr>
<tr><td>< 1.1+ | 6.1- ></td><td>2.50E-03</td></tr>
<tr><td>< 2.1+ | 7.1+ ></td><td>7.57E-02</td></tr>
<tr><td>< 2.1+ | 7.1- ></td><td>1.55E-02</td></tr>
<tr><td>< 3.1+ | 8.1+ ></td><td>3.80E-02</td></tr>
<tr><td>< 3.1+ | 8.1- ></td><td>3.88E-02</td></tr>
<tr><td>< 1.1+ | 7.1+ ></td><td>2.03E-02</td></tr>
<tr><td>< 1.1+ | 7.1- ></td><td>1.62E-03</td></tr>
<tr><td>< 2.1+ | 8.1+ ></td><td>1.78E-02</td></tr>
<tr><td>< 2.1+ | 8.1- ></td><td>7.05E-03</td></tr>
<tr><td>< 1.1+ | 8.1+ ></td><td>2.23E-02</td></tr>
<tr><td>< 1.1+ | 8.1- ></td><td>3.74E-03</td></tr>
</table>

5. References

[1] C. A. Gould, K. R. McClain, D. Reta, J. G. C. Kragskow, D. A. Marchiori, E. Lachman, E.-S. Choi, J. G. Analytis, R. D. Britt, N. F. Chilton, B. G. Harvey, J. R. Long, *Science* **2022**, *375*, 198.

[2] J. C. Vanjak, B. O. Wilkins, V. Vieru, N. S. Bhuvanesh, J. H. Reibenspies, C. D. Martin, L. F. Chibotaru, M. Nipp, *J. Am. Chem. Soc.* **2022**, *144*, 17743.

[3] F.-S. Guo, B. M. Day, Y.-C. Chen, M.-L. Tong, A. Mansikkamäki, R. A. Layfield, *Science* **2018**, *362*, 1400.

[4] A. H. Vincent, Y. L. Whyatt, N. F. Chilton, J. R. Long, *J. Am. Chem. Soc.* **2023**, *145*, 1572.

[5] K. R. McClain, C. A. Gould, K. Chakarawet, S. J. Teat, T. J. Groshens, J. R. Long, B. G. Harvey, *Chem. Sci.* **2018**, *9*, 8492.
